# Plectin dysfunction in neurons leads to tau accumulation on microtubules affecting neuritogenesis, organelle trafficking, pain sensitivity and memory

**DOI:** 10.1111/nan.12635

**Published:** 2020-06-25

**Authors:** R. G. Valencia, E. Mihailovska, L. Winter, K. Bauer, I. Fischer, G. Walko, J. Jorgacevski, M. Potokar, R. Zorec, G. Wiche

**Affiliations:** ^1^ Max F. Perutz Laboratories Department of Biochemistry and Cell Biology University of Vienna Vienna Austria; ^2^ Neuromuscular Research Department Center for Anatomy and Cell Biology Medical University of Vienna Vienna Austria; ^3^ Laboratory of Neuroendocrinology – Molecular Cell Physiology Faculty of Medicine Institute of Pathophysiology University of Ljubljana Ljubljana Slovenia; ^4^ Celica Biomedical Slovenia Ljubljana Slovenia; ^5^ Present address: Department of Immunology University Children’s Hospital Zurich Zurich Switzerland; ^6^ Present address: AFFiRiS AG Vienna Austria; ^7^ Present address: Department of Biology and Biochemistry University of Bath Bath UK

**Keywords:** axonal transport, microtubules, neuritogenesis, neurons, plectin, tau

## Abstract

**Aims:**

Plectin, a universally expressed multi‐functional cytolinker protein, is crucial for intermediate filament networking, including crosstalk with actomyosin and microtubules. In addition to its involvement in a number of diseases affecting skin, skeletal muscle, heart, and other stress‐exposed tissues, indications for a neuropathological role of plectin have emerged. Having identified P1c as the major isoform expressed in neural tissues in previous studies, our aim for the present work was to investigate whether, and by which mechanism(s), the targeted deletion of this isoform affects neuritogenesis and proper nerve cell functioning.

**Methods:**

For *ex vivo* phenotyping, we used dorsal root ganglion and hippocampal neurons derived from isoform P1c‐deficient and plectin‐null mice, complemented by *in vitro* experiments using purified proteins and cell fractions. To assess the physiological significance of the phenotypic alterations observed in P1c‐deficient neurons, P1c‐deficient and wild‐type littermate mice were subjected to standard behavioural tests.

**Results:**

We demonstrate that P1c affects axonal microtubule dynamics by isoform‐specific interaction with tubulin. P1c deficiency in neurons leads to altered dynamics of microtubules and excessive association with tau protein, affecting neuritogenesis, neurite branching, growth cone morphology, and translocation and directionality of movement of vesicles and mitochondria. On the organismal level, we found P1c deficiency manifesting as impaired pain sensitivity, diminished learning capabilities and reduced long‐term memory of mice.

**Conclusions:**

Revealing a regulatory role of plectin scaffolds in microtubule‐dependent nerve cell functions, our results have potential implications for cytoskeleton‐related neuropathies.

## Introduction

Plectin, a giant (>500 kDa) multifunctional cytolinker protein of universal occurrence, acts as a mechanical linker between the intermediate filament (IF) network and various cytoskeletal structures, including the subplasma membrane skeleton, a variety of specialized junctional complexes, Z‐disks of myofibres, mitochondria, and the nuclear lamina [1]. One of plectin's outstanding features is its functional versatility, which is primarily due to its multi‐domain structure enabling a broad range of interactions. Moreover due to an unusual transcript diversity that is largely based on at least nine different, relatively short N‐terminal sequences encoded by alternatively spliced first exons [2], different plectin isoforms are differentially targeted to distinct cellular locations where they function as universal IF‐docking sites. By recruiting and anchoring IF networks, along with a multitude of signalling and other structural proteins to these sites, plectin isoforms profoundly affect the microenvironment of the targeted structures and lead to cytoplasm compartmentalization.

In the absence of plectin, mechanically stressed cells lose their structural integrity, as observed in patients suffering from plectinopathies and in plectin‐deficient mice. The most common disease caused by mutations in the human plectin gene (*PLEC*) is epidermolysis bullosa simplex with muscular dystrophy (EBS‐MD), a rare autosomal skin blistering disorder associated with late‐onset muscular dystrophy. In addition to EBS‐MD, plectin mutations have been shown to cause EBS‐MD with myasthenic syndrome (EBS‐MD‐MyS), EBS with pyloric atresia (EBS‐PA), limb‐girdle muscular dystrophy type Q2 (LGMD2Q), and EBS‐Ogna [3]. A couple of EBS‐MD patients have been reported to display signs of a neurodegenerative disorder, suggesting that the expression of defective plectin may also interfere with the structural and functional integrity of the human central nervous system [4, 5]. Beyond that, very little is known about plectin’s involvement in neuropathology.

Plectin transcript profiling revealed that isoform P1c is a major isoform expressed in neural as well as in epidermal cells [2], both of which are ectoderm‐derived. Neural P1c was found to be expressed late in development and associated with postsynaptic dendrites of central nervous system neurons, spinal cord motor neurons, sciatic nerve axons and Schwann cells [6]. In adult mouse brain, P1c was found in all grey matter areas, especially in postsynaptic dendrites, and in all cortical layers of the cerebral cortex. The analysis of spinal cord sections highlighted that P1c is prominently expressed in both the central and peripheral nervous systems. Furthermore, the initial phenotypic analysis of a mouse line selectively lacking this isoform revealed reduced nerve conduction velocity in motor neurons combined with a shift from large diameter to small diameter axons [6]. Whether these alterations were physiologically significant, affecting essential brain functions and/or leading to pathological conditions, remained to be shown.

Based on an experimental approach that combined *ex vivo* studies of isoform P1c‐deficient (P1c^−/−^) primary dorsal root ganglion (DRG) and plectin‐null (P0) hippocampal neurons with *in vitro* experiments using recombinant proteins, we show here that the lack of P1c in neurons leads to alterations of MT dynamics and excessive association with tau protein. As a result, a number of fundamental neurite functions, including branching and translocation of vesicles and mitochondria, were found to be compromised, and on the organismal level, P1c^−/−^ mice showed impaired pain sensitivity and diminished mnemonic capabilities.

## Methods

### Animals

DRG neurons were isolated from 8‐ to 12‐week‐old C57BL/6J wild‐type (WT), P1c^−/−^ (Plec/Parp10^tm1.1Gwi^) [6], and P1b^−/−^ (Plec^tm6Gwi^) [7] mice, hippocampal neurons from new‐born WT and P0 (Plec^tm1Gwi^) [8] mice. Twelve‐week‐old male WT and P1c^−/−^ mice were used for behavioural analyses. All mice were inbred on a C57/BL6 background and backcrossed for 10 generations. Animals were fed *ad libitum* and housed in a temperature‐ and humidity‐controlled room in a specified pathogen‐free environment under 12:12 h light/dark cycles. All applicable international, national and institutional guidelines for the care and use of animals were followed. Animal studies were approved by the Federal Ministry for Science and Research (BMWF), Vienna, Austria (BMWF‐66.006/0028‐II/3b/2013).

### Cell culture

Primary DRG neurons were isolated from 8‐ to 12‐week‐old mice essentially as described in [9]. Neurons were collected in ice‐cold DMEM/F‐12 medium (Gibco, Waltham, MA, USA), centrifuged at 210 **g** for 2 min, and enzymatically dissociated in a mixture of 200 µl collagenase (4000 U/ml; Sigma‐Aldrich, St. Louis, MO, USA), 20 µl horse serum (Gibco), and 780 µl DMEM/F‐12 (per mouse) for 90 min at 37°C, followed by addition of 1 ml 0.05% trypsin (Gibco) supplemented with 1 µl DNase I (Sigma‐Aldrich) for 10–15 min at 37°C. Trypsinization was stopped by adding 2 ml DMEM/F‐12 containing 20% horse serum, and the neurons were carefully triturated through a narrowed Pasteur pipette several times. Cell suspensions were centrifuged at 120 **g** for 5 min, and cells resuspended in a mixture of 10 µl penicillin/streptomycin (5000 U/ml–5000 µg/ml; Gibco), 100 µl horse serum, 20 µl N‐2 supplement (Gibco), and 1870 µl DMEM/F‐12 (growth medium), and either processed for transfection, or directly plated onto glass coverslips that had been coated, first with poly L‐lysine (10 µg/ml in dH_2_O; Sigma‐Aldrich) overnight at 37°C, followed by laminin (10 µg/ml in PBS; Sigma‐Aldrich) for at least 3 h at 37°C. DRG neurons were cultivated at 37°C in a humidified atmosphere of 5% CO_2_.

For the preparation of primary hippocampal neurons from new‐born WT and P0 mice, hippocampi were isolated from dissected brain hemispheres in HBSS solution (Gibco) and dissociated in trypsin‐EDTA (Gibco) for 10 min at 37°C. Afterwards, the solution was replaced with 12 ml pre‐warmed HBSS and the hippocampi allowed to settle down by gravity. This washing step was repeated twice, before the HBSS solution was replaced with 4 ml DMEM (Gibco) containing 10% horse serum (plating medium). Hippocampi were carefully triturated through a narrowed Pasteur pipette for several times until they were completely dissociated. Cells were then plated onto coverslips and kept in plating medium for 1–2 h before exchanging the medium with neurobasal‐A growth medium (Gibco), supplemented with 2 mM L‐glutamine (Gibco) and 2% B27 serum‐free supplement (Gibco).

### Antibodies

The following primary antibodies were used for immunofluorescence microscopy (IFM) and/or immunoblotting (IB): isoform‐specific rabbit antiserum (AS) to P1c [6], rat monoclonal antibodies (mAbs) to α‐tubulin (clone YL1/2, SM2202P; Acris, Hiddenhausen, Germany), rabbit AS to tau (A‐0024; Dako, Glostrup, Denmark), mouse mAbs to α‐tubulin (clone B‐5‐1‐2, T5168; Sigma‐Aldrich), rabbit AS to GAPDH (G9545; Sigma‐Aldrich), mouse mAbs to GST (clone GST2, G1160; Sigma‐Aldrich), mouse mAbs to acetylated tubulin (clone 6‐11B‐1, T6793; Sigma‐Aldrich), rabbit AS to actin (A2066; Sigma‐Aldrich), rabbit AS to MAP1A/B [10], rabbit AS to MAP2 [10], rabbit AS to MAP1A light chain (LC) [11] and rabbit AS to MAP1B LC [12] (see also Table S1). For IFM, primary antibodies were used in combination with goat anti‐rat IgG Rhodamine red, goat anti‐rat IgG Alexa 488, goat anti‐mouse IgG Alexa 488, goat anti‐mouse IgG Cy5, goat anti‐rabbit IgG Alexa 488, goat anti‐rabbit IgG Cy5 (all from Jackson ImmunoResearch Laboratories, West Grove, PA, USA). For immunoblot analyses HRP‐conjugated secondary antibodies were used (Jackson ImmunoResearch Laboratories).

### Generation of cDNA constructs used for expression in bacteria and mammalian cells, and purification of recombinant proteins

For protein expression in bacteria, the following cDNA expression plasmids were generated by PCR: (i) plasmids encoding plectin‐GST (C‐terminal) fusion proteins corresponding to exons 1c‐8 (P1c‐8; amino acid residues 1–298, GenBank accession no AF188014), exons 1c‐30 (P1c‐30; 1–1374, NM 011117), exons 16–24 (P16–24; 603–989, NM 201394), and exons 20–21 (P20–21; 786–860, NM 201394); (ii) plasmids encoding untagged protein fragments of plectin corresponding to exons 1a‐8 (P1a‐8, amino acids 1–269, NM 201394), exons 1c‐8 (P1c‐8; 1–298, AF188014), exons 2–8 (P2–8; 67–298, AF188014), exons 2(2α3α)‐8 (P2(2α3α)‐8; 67‐315, AF188013), and exons 1c(2α3α)‐8 (P1c(2α3α)‐8; 1–315, AF188013). For expression of recombinant plectin fragments, cDNA constructs were cloned into expression vectors pGEX‐4T‐1 (GST‐tag; GE Healthcare, Chicago, IL, USA) or pGR66, a modified version of pBN120 without his‐tag [13]. Fusion and untagged proteins were expressed in *Escherichia coli* strain BL21 (DE3)_RIL_. GST fusion proteins were purified on glutathione sepharose 4B beads as described in the manufacturer’s instructions (GE Healthcare). Recombinant plectin fragments without tag were purified by FPLC [14]. Mammalian expression plasmids encoding full‐length mouse P1c, or truncated plectin versions P1c(2α3α)‐8 or P1f‐8 with C‐terminal EGFP tags have been described previously [15]. Plectin cDNA encoding protein fragments corresponding to P1c(2α3α)‐8, P1c(2α3α)‐32, P1c(2α3α)‐30, P16–24, P20–21 were excised from existing plasmids and inserted into the expression vector pEGFP‐N2 (BD Biosciences, San Jose, CA, USA). Mammalian expression plasmids encoding Fyn‐SH3 and EB3‐mCherry were kindly provided by Gloria Lee (Department of Internal Medicine, University of Iowa College of Medicine, USA), and Anna Akhmanova (Department of Biology, Faculty of Science, Utrecht University, the Netherlands), respectively.

### MT co‐sedimentation assays

For co‐sedimentation assays, highly purified (MAP‐free) porcine brain tubulin was prepared by phosphocellulose (PC) chromatography according to [16]. Prior to its in vitro assembly, PC‐purified tubulin in 100 mM Pipes, pH 6.8, 2 mM EGTA, and 1 mM MgCl_2_ (PEM) supplemented with 1 mM GTP was pre‐centrifuged at 45 000 **g** for 30 min at 4°C; and the protein content determined. To assemble MTs, 25 µM of PC‐purified tubulin were incubated in the presence of 10% (v/v) glycerol [17], 1 mM DTT, and 0.5% (v/v) Triton X‐100 for 1 h at 37°C. Recombinant plectin fragments (2.5 µM) were then added, mixtures were incubated for another 60 min, followed by sedimentation of MTs at 45 000 **g** for 30 min at 37°C. Prior to incubation with MTs, the recombinant proteins were desalted using spin columns equilibrated in PEM, followed by ultracentrifugation at 45 000 **g** for 30 min at 4°C. Supernatant and pellet fractions were separated by SDS‐PAGE and protein bands visualized by Coomassie blue staining. Gels were then scanned and the amounts of protein contained in individual bands were quantified using Quantiscan software.

### Fractionation of MT‐bound and ‐unbound proteins from brain and DRG cell lysates, and pull‐down assay

Dissected mouse brains were homogenized in 2 ml of 80 mM MES, 1 mM MgCl_2_, 2 mM EGTA, 30% glycerol, 0.1% Triton X‐100, 1 mM PMSF, and phosphatase inhibitor cocktails 1 and 3 (Sigma‐Aldrich) at 37°C, using a Dounce tissue grinder. After centrifugation at 7000 **g** for 2 min, a small aliquot of the supernatant was added to a 5:1 mixture of 62.5 mM Tris‐HCl, pH 6.8, 10% glycerol, 5% 2‐mercaptoethanol, 2.3% SDS, 1 mM EGTA, 1 mM EDTA, 1 mM PMSF, and phosphatase inhibitor cocktails 1 and 3 (solution 0+) and 500 mM Tris‐HCl pH 6.8, 600 mM DDT, 10% SDS, 0.1% bromophenol blue, and 30% glycerol (6× SDS sample buffer), and retained as lysate fraction, while the rest of the supernatant was centrifuged at 26 000 **g** for 20 min at 37°C. The supernatant (MT‐unbound fraction) was diluted with solution 0+ and 6× SDS sample buffer, while the pellet was resuspended in 100 mM MES, pH 6.5, 1 mM MgSO_4_, 1 mM EGTA, 2 mM DTT, 30% glycerol, 0.1% Triton X‐100, 1 mM PMSF, and phosphatase inhibitor cocktails 1 and 3, followed by dilution in solution 0+ and 6× SDS sample buffer (MT‐bound fraction). Lysates, supernatant and pellet fractions were subjected to SDS‐PAGE and IB. Alternatively, DRG neurons isolated from five mice were pooled and subjected to a similar procedure.

For pull‐down experiments of tau proteins present in cell lysates of adult mouse brain, brain tissue was snap‐frozen in liquid nitrogen, ground in a mortar, and homogenized in 80 mM MES, 1 mM MgCl_2_, 2 mM EGTA, 0.5% Triton X‐100, 30% glycerol, 1 mM PMSF, complete mini EDTA‐free protease inhibitor cocktail (Roche, Basel, Switzerland), and phosphatase inhibitor cocktails 1 and 3 (GST binding buffer) on ice using a Dounce homogenizer. Brain lysates were centrifuged at 14 000 **g** and 4°C for 5 min and pre‐cleared with glutathione sepharose 4B (GE Healthcare) for 1 h. Plectin‐GST fusion proteins were immobilized on glutathione sepharose in GST binding buffer for 30 min at 4°C, the beads were washed with ice‐cold PBS and then incubated with pre‐cleared brain lysates for 2 h at 4°C. Afterwards, they were washed twice with GST binding buffer, bound proteins were eluted by adding 6x SDS sample buffer, and input and eluate fractions assessed by SDS‐PAGE and IB.

### Transient transfection of DRG and hippocampal neurons

Primary DRG and hippocampal neurons were transiently transfected immediately after isolation (before plating) using the Amaxa basic neuron SCN Nucleofector kit (Lonza, Basel, Switzerland) according to the manufacturer’s instructions. Per transfection, 2 × 10^4^ cells were resuspended in 20 µl of basic neuron SCN Nucleofector solution and mixed with 0.5–1.0 µg of endotoxin‐free DNA. The resulting suspension was transferred to a Nucleofector cuvette and the SCN basic neuro program 6 was applied. Immediately after transfection, 500 µl of growth medium were added and the cuvette kept in an incubator at 37°C for 5 min, before carefully plating the cells onto pre‐coated coverslips.

### Preparation of DRG explants

To prepare explants, DRG neurons were isolated from adult mice and harvested in ice‐cold RPMI 1640 medium (Gibco) according to a protocol modified from [18] and [19]. Non‐separated cell aggregates were cut into halves that were placed onto drops (5–10 µl) of ice‐cold Matrigel TM (BD Biosciences) situated on coverslips. Finally, the specimens were completely covered with another drop of Matrigel.

### Fluorescence, phase contrast, and video microscopy

For IFM, isolated DRG and hippocampal neurons were fixed with 4% PFA (in 11% sucrose) for 30 min at 37°C, washed with PBS, and blocked with 5% BSA and 0.1% Triton X‐100 (in PBS) for 1 h at room temperature. After blocking, cells were incubated with primary antibodies diluted in 5% BSA (in PBS) for 3 h at room temperature in a humidified chamber. After washing (3 × 5 min) with PBS, cells were incubated with secondary antibodies (diluted in PBS) for 2 h at room temperature, washed (3 × 5 min) with PBS, and mounted in Mowiol 4‐88 (Sigma Aldrich). For visualization of actin, cells were stained with Texas Red‐X (T‐7471) phalloidin (Molecular Probes, Eugene, OR, USA). Confocal microscopy was performed using a fluorescence laser scanning microscope (Zeiss LSM710, or LSM700 ; Zeiss, Oberkochen, Germany) equipped with a Plan‐Apochromat 63 × 1.4 NA objective lens. Images were recorded using the LSM510 or LSM710 module, respectively, and the Zeiss ZEN software.

DRG explants were recorded using an AxioObserver Z1 microscope equipped with phase contrast optics and an AxioCamMRm (Zeiss) digital camera, at 37°C in a humidified atmosphere of 5% CO_2_. Frames were taken with Plan‐Apochromat 40 × 0.85 NA or LD A Plan 32 × 0.4 NA objective lenses. Images were acquired with Zeiss AxioVision 4.8.1. software and further analysed with ImageJ software (US. National Institute of Health, Bethesda, MD, USA) for manual tracking.

For visualizing MT dynamics, DRG neurons expressing EB3‐mCherry were kept in a closed POCmini cultivation system (Zeiss) at 37°C in a humidified atmosphere of 5% CO_2_. Live‐cell imaging was performed using an Inverse Live Spinning Disk/Nanodissection unit (1.318), equipped with an Evolve EM‐CCD camera, and an EC Plan‐Neofluar 100×/1.3 Oil Iris objective. Frames were collected every 2 s during 5 min. Individual comets were traced using Metamorph 6.3 software (MDS Analytical Technologies, Sunnyvale, CA, USA).

For analysing mitochondrial dynamics, DRG neurons were incubated with 0.1 µM MitoTracker red CMXRos (Thermo Fischer Scientific, Waltham, MA, USA) in growth medium for 15 min before being processed for video microscopy. Videos were recorded using a similar set up as for monitoring MT dynamics, except for using a Plan‐Apochromat 63×/1.4 Oil DIC objective (Zeiss).

### Monitoring of synaptic vesicles

Vesicles were labelled with LysoTracker™ Red DND‐99 dye (37°C, 5 min, 200 nM; Thermo Fisher Scientific), then the samples were briefly washed and imaged in the extracellular solution (119 mM NaCl, 2.5 mM KCl, 2 mM CaCl_2_, 2 mM MgCl_2_, 30 mM glucose, 25 mM Hepes, pH 7.3). Time lapse recordings were acquired at 32°C with a laser confocal microscope (LSM 780; Zeiss) using an oil‐immersion objective (63×/NA 1.4). Lysotracker dye was excited by a DPSS laser (561 nm), and the emission light was filtered with a bandpass filter at 567–649 nm. Images were acquired every 2 s for 4 min.

Analysis of vesicle mobility was performed by ParticleTR software (Celica Biomedical, Ljubljana, Slovenia), as described [20]. Briefly, the software directly fits two‐dimensional (2D) Gaussian curves to the vesicle intensity profile, and the peak of the Gaussian 2D curve is recorded as the coordinates of the vesicle in each frame. The coordinates of vesicle positions enable calculations of vesicle mobility parameters, here presented as speed.

### Behavioural assessments of mice

For behavioural analyses, 12‐week‐old male B57BL/6 (WT) and P1c^−/−^ mice [6] were tested during 5 weeks in a battery of neurocognitive tests:

- Pain sensitivity of mice was assessed using the hot plate test according to [21]. Mice were placed on a thermally controlled metal plate (52–55°C) and the time between placement of a mouse on the plate and licking, lifting, or shaking of a hind paw and/or jumping was recorded as discomfort response index. Mice were removed from the hot plate immediately after their first response.
- To assess fear conditioned memory, mice were first subjected to a conditional training, which consisted of placing them into the test chamber for 2 min for habituation, then exposing them twice to a mild foot shock (1 s, 0.5 mA) co‐terminated with an auditory cue (85 dB, 10 kHz) with 2 min inter‐trial intervals. The time (s) spent freezing (immobility for at least 2 s) in the test chamber during the conditional training was recorded and analysed using FreezeFrame and FreezeView software (Actimetrics, Wilmette, IL, USA), respectively. For assessing contextual learning, the mouse was returned 24 h later into the same chamber in the absence of foot shock and cue, and scored for freezing behaviour every 2 s for 4 min. Thus, the time spent freezing in the same chamber measured the contextual conditioned fear. To assess cue conditioning, 5 h after the contextual test, the test chamber was altered (different floor, wall texture, lightning, and odour) and the mice were exposed to the auditory cue. The time spent freezing in the chamber was taken as a measure for cue conditioned fear. Fear conditioning was assessed by comparing the freezing behaviour in the absence of the cue (2 min habituation; no stimulus) with that in the presence of the cue (2 × 1 min, 1 min pause in between; with stimulus). Three weeks after the conditional training, both tests were repeated to assess long‐term memory.
- In the Morris water maze test, performed according to [22], the animals had to swim in water (27°C or colder) to find a hidden platform (1 cm beneath the water surface) using visual cues (placed on the walls of the pool). Escape from the water was used as positive reinforcement. The test consisted of 5 days of acquisition (learning), and two consecutive trials immediately after (short‐term memory) and 72 h (long‐term memory) after finishing the acquisition phase. One day before the acquisition phase, a visible platform test was performed to exclude motor and visual acuity impairment. During the acquisition phase (day 1–5), mice were tested in the hidden platform version of the test. The platform was marked with a flag, and the animals were gently placed in the water in any of the four quadrants of the pool and allowed to swim for 1 min to find the platform. If they found it, they were allowed to sit on it for 5 s, before they were removed to their home cage. If they did not find the platform after 1 min, the animals were placed on the platform for 5 s before they were placed back in their home cage. Each subject was given eight training trials per day (four times in the morning, four times in the evening). For both trials, immediately after and 72 h after the acquisition phase, the platform was removed. The animals were placed in the water and allowed to swim for 1 min. Each trial began by placing the mouse in the water, near and facing the wall of the pool starting from the SW side. Position, tracking length, swim speed and latency in finding the platform were recorded automatically using the Top Scan software (CleverSysInc^©^, Reston, VA, USA) to analyse whether the mice remained in the area where the platform has been placed before.

### Quantification and statistical analysis

Data analyses and statistical evaluations were performed using Excel 2010 (Microsoft, Redmond, WA, USA) and SPSS Statistics v.19 (IBM, Armonk, NY, USA). The number of experiments is indicated in the figure legends; data are given as mean ± SEM or mean ± SD. Comparisons between values of two groups were made using an unpaired, two‐tailed Student’s *t* test (α = 0.05) or Wilcoxon–Mann–Whitney *U*‐test. Comparisons among values of multiple groups were performed using one‐way analysis of variance (ANOVA; α = 0.05). The significance between values of individual groups and controls was subsequently determined using Tukey’s *posthoc* test. *P*‐values are *<0.05, **<0.01, and ***<0.001; a *P*‐value <0.05 was considered statistically significant. In general, three independent experiments were evaluated. Final assembly and preparation of figures was undertaken using Adobe Illustrator CC 2015.

## Results

### Isoform‐specific association of P1c with MTs of DRG and hippocampal neurons

**Figure 1:**
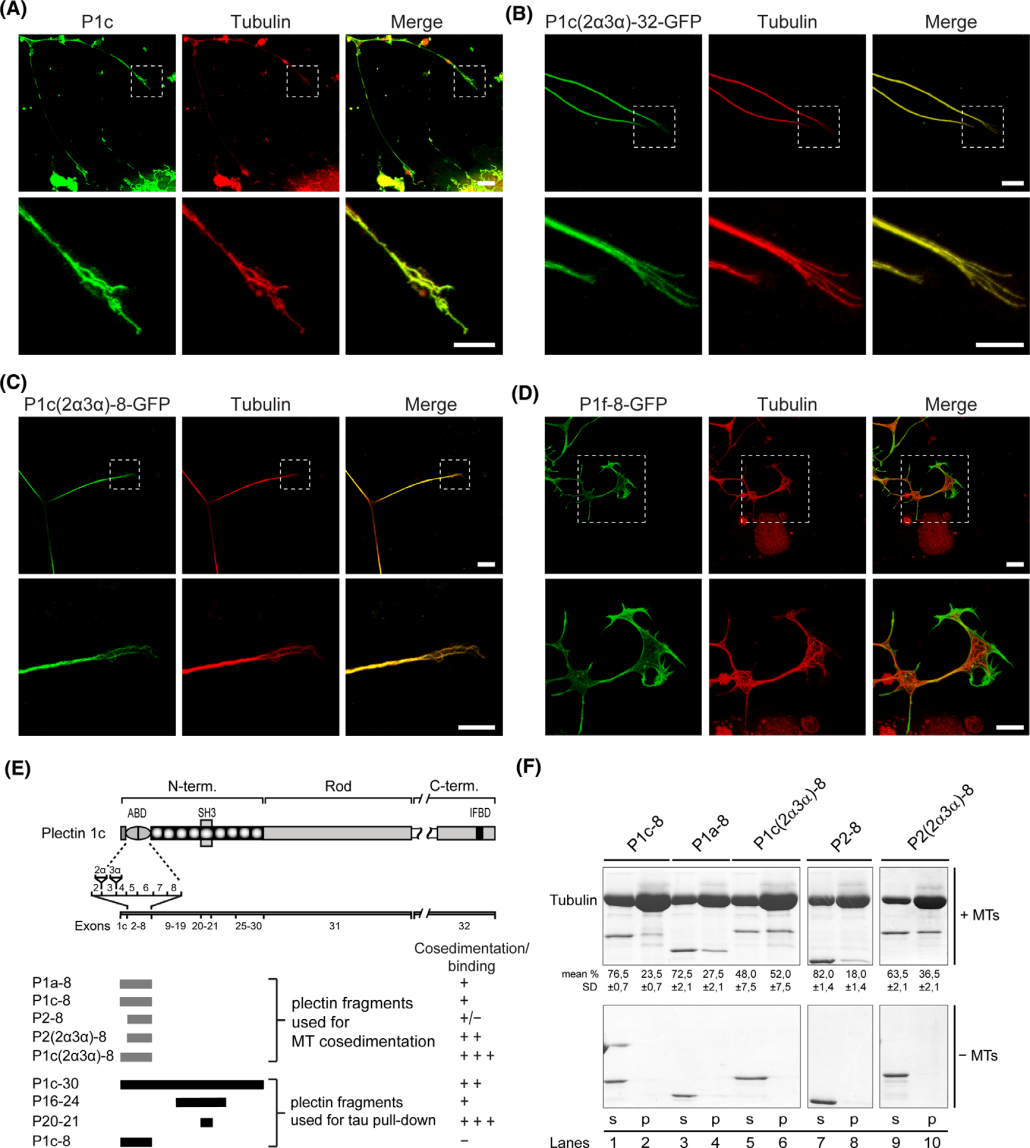
*In vivo* and *in vitro* association of plectin isoform P1c with neuronal MTs. (**A**) Double immunostaining of dorsal root ganglion (DRG) neurons isolated from a WT mouse using isoform P1c‐specific and anti‐tubulin antibodies. Lower panels are magnifications of boxed areas indicated in upper panels. Scale bars: 10 µm (upper panels), 5 µm (lower panels). Note that endogenous P1c shows extensive co‐localization with tubulin in axons as well as in growth cones. (**B**–**D**) WT DRG neurons were transfected with expression plasmids encoding C‐terminally green fluorescent protein‐tagged versions of full length P1c (P1c(2α3α)‐32) (**B**), truncated P1c (P1c(2α3α)‐8) (**C**), or truncated P1f (P1f‐8) (**D**). Shown are confocal images of transfected neurons (in green), additionally immunolabelled for tubulin (in red). Boxed areas in upper panels are shown at higher magnification in the respective lower panels. Scale bars: 10 µm (upper panels), 5 µm (lower panels). (**E**) Schematic representation of plectin subdomains, exon allocations, and plectin fragments used for co‐sedimentation and pull‐down assays. N‐ and C‐term, N‐ and C‐terminal domains; actin binding domain, SH3, and IFBD, actin‐, MAP‐ and intermediate filament‐binding domains, respectively. Results from co‐sedimentation and pull‐down assays are summarized in the last column. (**F**) Co‐sedimentation of truncated plectin fragments with MTs. After incubation of plectin fragments with (+) or without (−) MTs and centrifugation, pellet (p) and soluble (s) fractions were subjected to SDS‐PAGE and Coomassie‐stained bands densitometrically quantified. The relative amounts of plectin fragments (percentages) found in both fractions are given as means ± SD (*n* = 3) underneath gel bands of upper panel.

Having previously shown that the lack of P1c in keratinocytes affects MT dynamics and leads to alterations of MT‐dependent cellular functions [23], we investigated whether P1c played a similar role in neurons, where tightly controlled MT network organization and dynamics are essential for proper cell functioning. First, we assessed P1c co‐localization with MTs by subjecting primary DRG neurons to double immunolabelling using isoform‐specific antibodies to P1c and antibodies to α‐tubulin. As shown in Figure 1**A** P1c‐tubulin co‐localization was found along MTs in the shaft of neurites as well as in their growth cones. Assessing MT‐targeting of P1c in an alternative way, DRG neurons were transfected with expression plasmids encoding green fluorescent protein (GFP)‐tagged fusion proteins of either full length P1c (P1c(2α3α)‐32‐GFP), or a truncated version of P1c encoded by exons 1c‐8 (P1c(2α3α)‐8‐GFP), both containing neural P1c‐specific sequences encoded by the differentially spliced exons 2α and 3α [2]. In addition, as a control for isoform specificity, DRG neurons were transfected with a plasmid encoding a similar truncated version (P1f‐8‐GFP) of P1f, an isoform generally associated with the plasma membrane. As shown in Figure 1**B**,**C**, both, the full‐length as well as the truncated versions of P1c showed co‐localization with MTs, whereas the distribution of P1f‐8 was distinguishable from MTs, especially in peripheral growth cone areas (Figure 1**D**). These observations confirmed that the recruitment of P1c to neuronal MTs was isoform‐specific.

To investigate whether P1c interacts with MTs via direct binding to tubulin, and to characterize the molecular interfaces involved, we subjected a series of recombinant N‐terminal fragments of plectin (see Figure 1**E**) to MT co‐sedimentation assays using tubulin purified and freed of MT‐associated proteins (MAPs) by phospho‐cellulose chromatography. To achieve polymerization without MAPs, the concentration of tubulin was kept high (5 mg/ml) and the assay was performed in the presence of glycerol. It was of particular interest to test whether the actin binding domain (ABD) of the P1c variant expressed exclusively in nerve tissues, containing additional short sequences of 5 and 12 amino acid residues (encoded by alternatively spliced exons 2α and 3α, respectively; [2]), had any effect on MT interaction. When fragments P1c‐8 and P1a‐8, both containing isoform‐specific first exon‐encoded plectin sequences preceding the ABD (without exon 2α‐ and 3α‐encoded sequences), and fragment P2‐8 (comprising the corresponding ABD alone) were tested, their co‐sedimentation with MTs was relatively weak, as revealed by quantitative analysis of MT fractions (Figure 1**F**, lanes 1–4,7,8). However, when fragment P1c(2α3α)‐8, corresponding to the unique brain variant, was used, a large portion of it (52.0%) co‐sedimented with MTs (lanes 5, 6). P2(2α3α)‐8, a corresponding fragment without the preceding exon 1c‐encoded sequence, co‐sedimented less efficiently (36.5%) (lanes 9, 10), yet still at a higher rate than the fragments P2‐8, P1c‐8, and P1a‐8. These results demonstrated that the P1c‐specific sequence combination encoded by exons 1c‐8, including 2α and 3α, formed an optimal binding domain for MAP‐free tubulin polymers. Both, its *in vivo* association with MTs in neurons and its *in vitro* binding to MTs, classified P1c as a MAP.

### P1c‐deficiency in neural cells leads to elevated levels of MT‐bound tau

**Figure 2:**
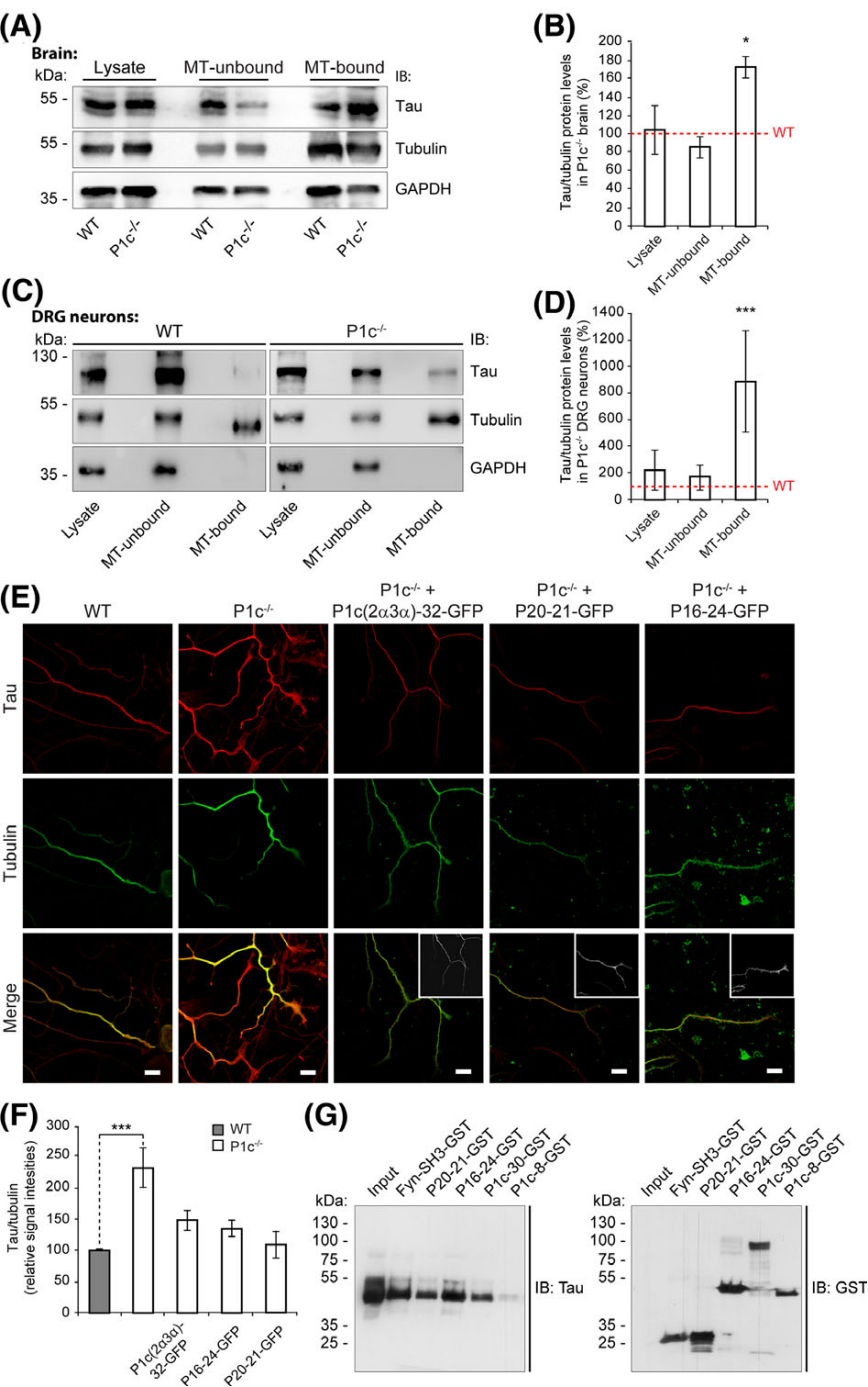
Plectin‐dependent association of tau protein with neuronal MTs assessed by co‐fractionation, *in situ* localization and pull‐down. (**A**,**C**) Cell lysates prepared under MT‐stabilizing conditions from WT and P1c‐deficient mouse brain (**A**) or dorsal root ganglion (DRG) neurons (**C**) were fractionated, and total cell lysates, MT‐unbound, and MT‐bound fractions were analysed by immunoblotting using antibodies as indicated. (**B**,**D**) Tau signal intensities shown in (**A**) and (**C**) normalized to tubulin levels. Mean ± SEM (*n* = 3). *P < 0.05 and ***P < 0.001 compared with WT; unpaired Student’s *t* test. (**E**) Confocal fluorescence microscopy of anti‐tubulin (combined with anti‐rat Rhodamine Red, here depicted in green) and anti‐tau (combined with anti‐rabbit Cy5, here depicted in red) double immunolabelled WT and P1c^−/−^ DRG neurons, and P1c^−/−^ DRG neurons transfected with expression plasmids encoding proteins fused (C‐terminally) to green fluorescent protein (GFP). Insets, transfected neurons identified by GFP‐specific signals (shown in black and white). Scale bars: 10 µm. Note: (i) enhanced tau signals in axons of P1c^−/−^ compared to WT neurons, and (ii) reduced tau‐specific signal intensity upon transfection with any one of the three plectin cDNA constructs tested. (**F**) Statistical evaluation of tau‐specific signal intensities normalized to tubulin levels (all signals were recorded below saturation levels). Mean ± SEM [axons analysed: WT (*n* = 105), P1c^−/−^ (*n* = 93), P1c^−/−^ + P1c(2α3α)‐32 (*n* = 44), P1c^−/−^ + P16–24 (*n* = 101), P1c^−/−^ + P20–21 (*n* = 51)]. ***P < 0.001 compared with WT; one‐way ANOVA and *post‐hoc* Tukey correction. Note: (i) there is an increase (by a factor of 2.3) in the tau/tubulin ratio in P1c^−/−^ compared to WT neurons, and (ii) transfection of P1c^−/−^ neurons with full‐length P1c or N‐terminal plectin fragments P20–21 and P16–24 largely restored tau signals to WT control levels. (**G**) Pull‐down of tau (~55 kDa) from brain lysates using GST‐fusion proteins as indicated. GST‐tagged proteins, immobilized on glutathione‐sepharose beads, were incubated with mouse brain lysates (input), and input and eluate fractions subjected to immunoblotting using antibodies to tau and GST. Note that pull‐down of tau was observed with Fyn‐SH3 (positive control) as well as plectin fragments P20–21, P16–24, and P1c‐30; pull‐down with P1c‐8 was negligible.

Tau protein, one of the major neuronal MAPs, has long been considered to play a crucial role in stabilizing axonal MTs and in regulating axonal transport [24, 25]. Recently, however, it has been suggested that tau, rather than stabilizing MTs, enables MTs to have long labile domains, favouring their dynamics [26]. To investigate whether the absence of neuronal P1c affects tau’s association with endogenous MTs, we quantified MT‐bound tau in brain lysates prepared from WT and plectin‐deficient mice under conditions where the polymeric state of MTs is preserved [27]. When MTs and their co‐assembling (bound) proteins were sedimented by high‐speed centrifugation and analysed by IB, along with total lysates and the MT‐unbound (soluble) fractions, the level of the major ~50 kDa tau isoform expressed in brain was found to be increased by a factor of 1.7 in P1c^−/−^ compared to WT samples (Figure 2**A**,**B**), with the levels of tubulin remaining largely unchanged. Compatible results were obtained when DRG neurons, instead of brain, were analysed in a similar way. In this case, the relative amount of a tau isoform of ~120 kDa, that is predominantly expressed in this type of neurons [28], was found to be drastically increased (8.9‐fold) in MT‐bound fractions of P1c^−/−^ compared to WT neurons (Figure 2**C**,**D**). In addition, we comparatively measured MT association of several other genuine MAPs present in brain lysates from WT and P1c‐deficient mice, among them MAP1A/B, MAP2, MAP1A LC, and MAP1B LC. As shown in Figure S1, none of these MAPs showed an increase in MT association in the absence of P1c, but rather a decrease (MAP1A LC, MAP1B LC) or no change (MAP1A/B, MAP2), supporting the notion that accumulation on MTs was specific for tau.

Immunolabelling of WT and P1c^−/−^ DRG neurons for tau and tubulin revealed both proteins to co‐localize along the axons of either cell type (Figure 2**E**), indicating that P1c deficiency did not grossly influence tau localization. However, the tau‐specific fluorescence signals appeared to be stronger in axons of P1c^−/−^ DRG neurons than in WT cells. The quantification of signal intensities revealed a 2.3‐fold higher tau to tubulin ratio in P1c^−/−^ DRG neurons (Figure 2**F**). To assess whether P1c deficiency was directly linked to and causative of elevated levels of MT‐bound tau, we performed rescue experiments where P1c^−/−^ DRG neurons were transiently transfected with cDNA expression plasmids encoding GFP fusion proteins that corresponded either to full‐length P1c (P1c(2α3α)‐32‐GFP), or to one of two protein fragments (P16–24‐GFP or P20–21‐GFP) that both contained the plectin SH3 domain (with/without flanking sequences, see Figure 1**E**), previously identified as the MAP‐binding domain of plectin [23]. As shown in Figure 2**E**,**F**, expression of any of these plasmids led to partial phenotype rescue, with P20–21‐GFP being most effective. When in similar experiments WT and P0 hippocampal neurons were stained for tau and tubulin, again, an enhanced fluorescence signal for tau was detected in axons of plectin‐deficient neurons (Figure S2**A**), amounting to an elevation of tau protein levels to 174% of WT levels (Figure S2**B**). In this case, the forced expression of full length P1c in P0 hippocampal neurons led to a reduction of tau‐specific fluorescence levels to even below those of WT cells (Figure S2**B**). These observations indicated that increased tau protein association with axonal MTs, as a result of plectin deficiency, was a general phenomenon applicable to neurons of both, the central (hippocampus) and the peripheral (DRG) nervous system.

To demonstrate P1c‐tau interaction *in vitro*, we used recombinant N‐terminal fragments of P1c (expressed as N‐terminally GST‐tagged fusion proteins) for a pull‐down assay of endogenous tau proteins present in mouse brain lysates. The plectin fragments used (see Figure 1**E**) included P1c’s N terminus extending to the rod domain (P1c‐30), the N terminus extending to the ABD domain (P1c‐8), a central part of P1c’s N‐terminal domain containing the SH3 domain and its flanking spectrin repeat sequences (P16–24), as well as the SH3 domain alone (P20–21); in addition, the SH3 domain of the non‐receptor tyrosine kinase fyn (Fyn‐SH3), which has been shown to interact with tau [29], was used as a positive control. As shown in Figure 2**G**, all of the plectin fragments containing the SH3 domain as well as Fyn‐SH3 showed robust binding to endogenous tau protein. In contrast, fragment P1c‐8, comprising the ABD but not the SH3 domain, showed comparatively weak and hardly specific binding, confirming that the SH3 domain was required for efficient binding to tau.

### P1c‐deficiency affects axonal MT dynamics, neuritogenesis and neuronal morphology

**Figure 3:**
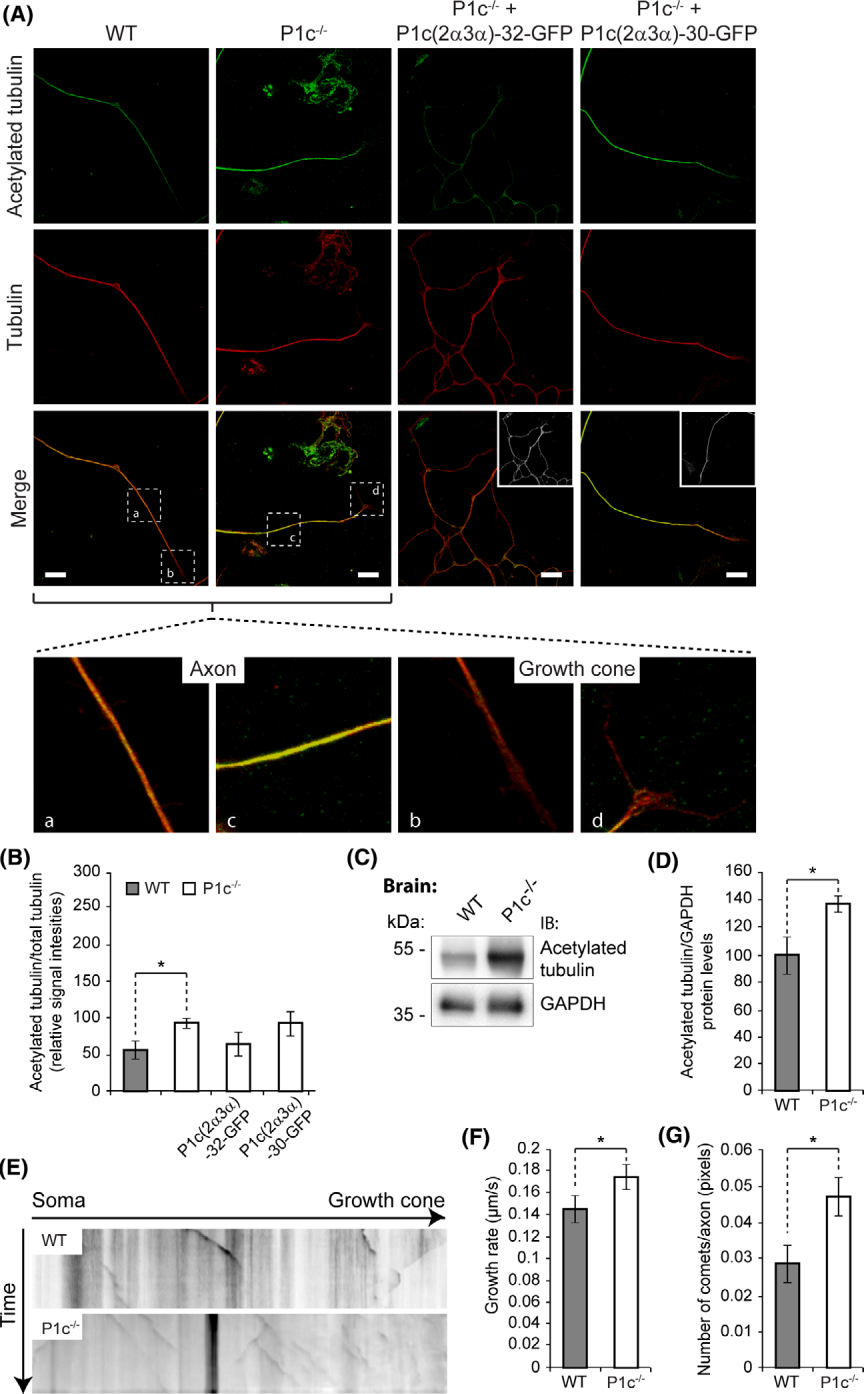
P1c‐deficiency affects the acetylation status and the dynamics of axonal MTs. (**A**) IFM of WT and P1c^−/−^ dorsal root ganglion (DRG) neurons using antibodies to acetylated tubulin (combined with anti‐mouse Cy5, here depicted in green) and total tubulin (combined with anti‐rat Rhodamine Red, here depicted in red). The first two columns show untransfected cells, the rest P1c^−/−^ neurons transfected with expression plasmids encoding C‐terminally green fluorescent protein (GFP)‐tagged fusion proteins as indicated. Boxed axonal and growth cone areas (a–d) in row merge (first two columns) are shown magnified in bottom row. Insets (row merge, last two columns), transfected cells identified by GFP‐specific signals (shown in black and white). Note, the axonal shafts, as depicted in (a) and (c), contain increased amounts of acetylated MTs in P1c^−/−^ compared to WT neurons, while labile MTs in the growth cones appear non‐acetylated in both cell types (b,d). Scale bars: 4 µm. (**B**) Statistical evaluation of signal intensities of acetylated tubulin normalized to those of total tubulin, based on data as shown in (**A**). Mean ± SEM [WT (*n* = 23), P1c^−/−^ (*n* = 19), P1c^−/−^ + P1c(2α3α)‐32 (*n* = 30), P1c^−/−^ + P1c(2α3α)‐30 (*n* = 18)]. *P < 0.05 compared with WT; one‐way ANOVA and *post‐hoc* Tukey correction. (**C**) Cell lysates prepared from WT and P1c‐deficient mouse brain were analysed by immunoblotting using antibodies as indicated. (**D**) Acetylated tubulin signal intensities shown in (**C**) normalized to GAPDH levels. Mean ± SEM (*n* = 3). (**E**) Representative time‐lapse recordings of EB3‐mCherry‐expressing WT and P1c^−/−^ DRG neurons converted into kymographs showing the projection of EB3‐positive comet movements over time (*y*‐axis). Moving comets are represented by diagonal, stationary comets by straight lines. Note: (i) the number of EB3‐mCherry comets in P1c^−/−^ is increased compared to WT neurons, and (ii) within the same period of time, the EB3‐mCherry comets traced in P1c^−/−^ DRG neurons cover longer tracks than the ones in WT cells. (**F**,**G**) Statistical analyses of MT growth rates (**F**) and number of comets per axon (**G**) evaluated from time‐lapse recordings as in (**E**). Mean ± SEM [WT (*n* = 22), P1c^−/−^ (*n* = 32)]; independent experiments (*n* = 4). *P < 0.05 compared with WT; unpaired Student’s *t* test (**D**,**F**,**G**).

In living cells, a certain proportion of the polymerized MT pool is stabilized, while the labile fraction of MT fibres displays rapid dynamics [30]. As posttranslational acetylation of α‐tubulin is usually associated with MT stabilization [30, 31], we evaluated acetylated tubulin‐positive regions *vs*. unmodified (total) tubulin‐positive regions in WT and P1c^−/−^ neurons using semi‐quantitative IFM and antibodies that recognized only acetylated, or both tubulin versions (Figure 3**A**). Interestingly, we observed statistically 1.6 times higher levels of acetylated tubulin in P1c^−/−^ compared to WT neurons (Figure 3**A**,**B**). It appeared, however, that this applied mainly to P1c‐deficient axonal areas, while in growth cone areas, the levels of acetylated tubulin were comparable (Figure 3**A**, magnified panels b and d). In a similar analysis of hippocampal neurons isolated from new‐born P0 mice [8], we found 2.2 times higher levels of acetylated tubulin compared to corresponding WT cells (Figure S3**A**,**B**). Likewise, when the amount of acetylated tubulin was quantitatively assessed in P1c^−/−^ brain lysates using IB, 1.4 times higher levels compared to WT tissue were found (Figure 3**C**,**D**). Moreover in rescue experiments, where P1c^−/−^ DRG neurons were transiently transfected with cDNA expression plasmids encoding GFP fusion proteins of either full‐length P1c (P1c(2α3α)‐32‐GFP), or a truncated version (P1c(2α3α)‐30‐GFP) lacking plectin’s rod and C‐terminal IF‐binding domain [13] (see also Figure 1**E**), only the expression of full‐length P1c, but not of the truncated fragment led to a phenotype reversal (Figure 3**A**,**B**). In all, these data are consistent with the hypothesis that stable (not tau‐associated) MT domains on axonal MTs become more acetylated as a result of plectin deficiency, probably increasing their stability.

To assess whether P1c deficiency affected dynamic properties of the labile domains of neuronal MTs, we performed video microscopy of WT and P1c^−/−^ DRG neurons expressing mCherry‐tagged EB3, a MT plus‐end tracking protein (+TIP) which is part of the MT elongation machinery, but is absent from non‐growing or disassembling MT ends [32]. Kymographs of time‐lapse images showed not only a near 1.6 times higher number of EB3‐positive comets, but also an increase to ~120% in comet velocity (distance covered within time period) in P1c^−/−^ compared to WT DRG neurons (Figure 3**E**–**G**). Higher growth rates and frequency of MT plus‐ends implied that overall the labile proportion of the MT network was more dynamic, and probably even more labile [30], in the absence of P1c.

**Figure 4:**
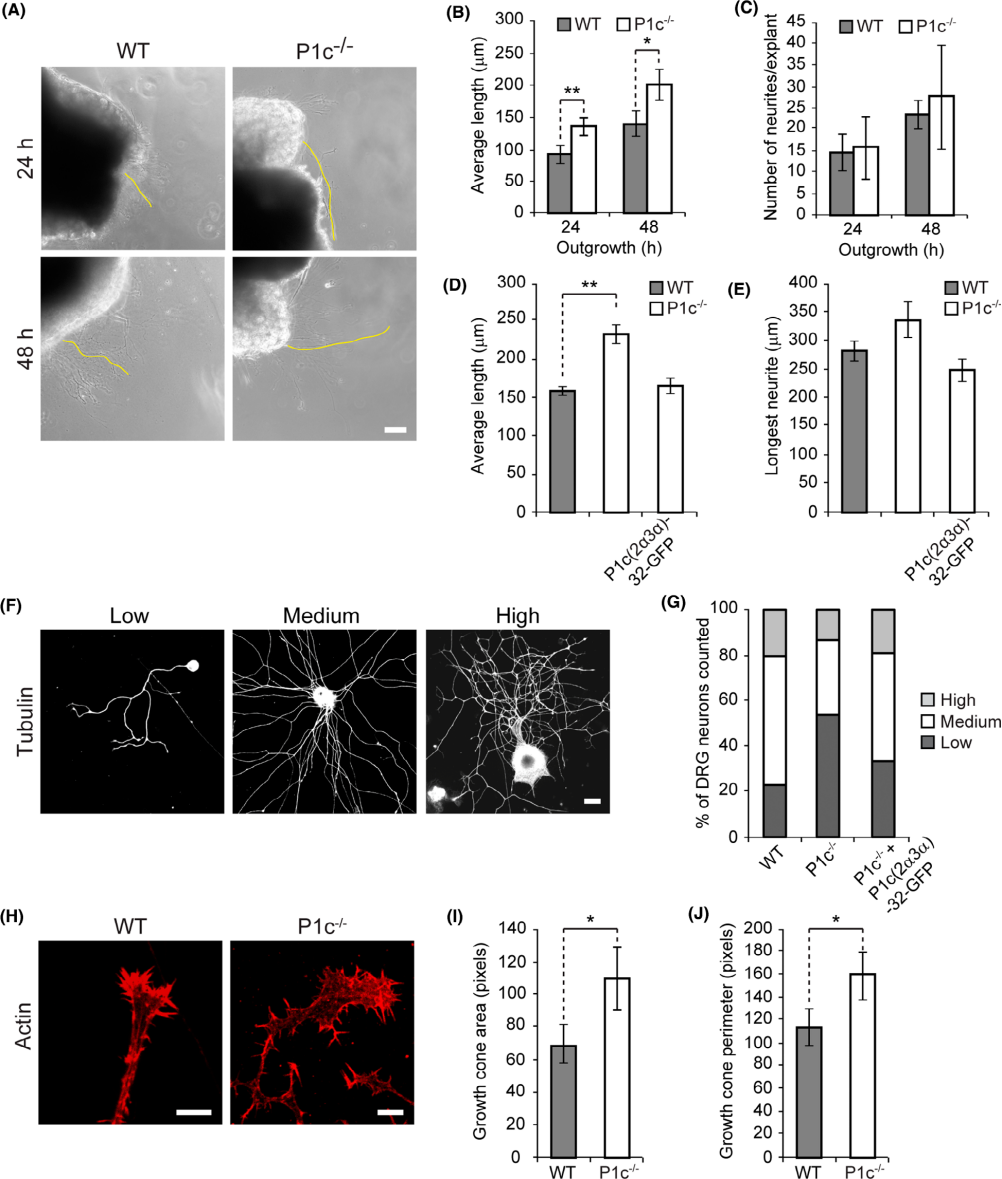
P1c‐deficiency affects neuritogenesis, neurite branching, and growth cone morphology. (**A**) Dorsal root ganglion (DRG) explants derived from WT and P1c^−/−^ mice were cultivated in Matrigel and representative phase contrast images of the outgrowing neurites were taken after plating at time points indicated. Yellow outlining, longest neurite visualized. Scale bars: 65 µm. (**B**,**C**) Statistical evaluation of the average neurite length (**B**), and the total number of neurites (**C**) per explant. Mean ± SEM [WT (*n* = 59), P1c^−/−^ (*n* = 6)]. (**D**,**E**) Statistical evaluation of the average neurite length (**D**), and the length of longest neurite (**E**) of transfected/untransfected DRG neurons. Mean ± SEM [WT (*n* = 155), P1c^−/−^ (*n* = 102), P1c^−/−^ + P1c(2α3α)‐32 (*n* = 37)]. (**F**) Classification of DRG neuron morphology according to the number of branching points. Low, medium, and high correspond to <1.5, 1.5–3.0, and >3.0 branching points per neurite length (measured in pixels × 10^2^), respectively. Scale bar: 20 µm. (**G**) Statistical evaluation of branching points identified in untransfected WT and P1c^−/−^ DRG neurons, and in P1c^−/−^ neurons transfected with an expression plasmid encoding green fluorescent protein (GFP)‐tagged full‐length P1c. [WT (*n* = 132), P1c^−/−^ (*n* = 108), P1c^−/−^ + P1c(2α3α)‐32 (*n* = 27)]. Note the shift from low to medium, and medium to high level branching in transfected cells. (**H**) Growth cones of WT and P1c^−/−^ DRG neurons visualized by labelling of actin with fluorescent phalloidin. Scale bars: 5 µm. (**I**,**J**) Statistical evaluation of growth cone areas (**I**) and perimeters (**J**). Mean ± SEM (*n* = 30 each). *P < 0.05 and **P < 0.01 compared with WT; unpaired Student’s *t* test (**B**–**E**,**I**,**J**).

Providing part of the machinery that executes neuronal branching and neurite extension, MTs play an essential role in neuritogenesis [33]. To investigate whether P1c deficiency has any influence on neuronal development, DRG explants derived from WT and P1c^−/−^ mice were cultivated in Matrigel and neurite outgrowth was measured after 24 and 48 h (Figure 4**A**). Interestingly, at both time points, neurites from P1c^−/−^ explants were significantly longer than those of WT neurites (Figure 4**B**), while regarding the number of neurites formed, the two genotypes showed no significant differences (Figure 4**C**). Similar to explants, individually isolated P1c^−/−^ DRG neurons exhibited increased average neurite length (Figure 4**D**), while the longest observed neurites per explant were not significantly altered (Figure 4**E**). When P1c^−/−^ DRG neurons were transfected with expression plasmids encoding GFP‐tagged fusion proteins of full‐length P1c (P1c(2α3α)‐32‐GFP), neurite lengths were restored to levels similar to that of WT cells (Figure 4**D**,**E**). These data indicated that the observed phenotype was indeed isoform P1c‐specific and directly linked to P1c deficiency.

To assess the frequency of neurite branching events, DRG neurons were classified according to the number of their branching points normalized to neurite length. On a scale defined in Figure 4**F**, the majority (57%) of WT neurons showed branching at a medium, 23% at a low, and 20% at a high level. In contrast, in P1c‐deficient neurons, low‐level branching was most frequent (54%), medium‐level branching was underrepresented (33%), and high‐level branching drastically reduced (13%) (Figure 4**F**,**G**). Forced expression of full length P1c (P1c(2α3α)‐32‐GFP) in P1c^−/−^ DRG neurons led to restoration (rescue) of their branching phenotype to levels comparable to those of WT cells (33% low, 48% medium, and 19% highly branched neurons) (Figure 4**G**). Likewise, when hippocampal neurons isolated from P0 mice were classified in a similar way, the proportions of low‐ and medium‐level branching were found increased (from 28% to 37% and from 24% to 52%, respectively), while high‐level branching was strongly reduced (from 48% to 11%) compared to WT neurons (Figure S4**A**,**B**).

P1c‐deficient DRG neurons differed from their WT counterparts also in growth cone morphology, as visualized by phalloidin‐labelling of their actin cytoskeleton (Figure 4**H**). Compared to WT, P1c^−/−^ growth cones appeared more spread out, i.e. were larger, and exhibited more protrusions, manifesting as a 1.6‐fold larger area covered, and 1.4‐fold larger perimeters (Figure 4**I**,**J**). Similar differences in morphology were observed for growth cones of hippocampal neurons derived from new‐born WT and P0 mice (Figure S4**C**). In this case, the growth cones of mutant neurons showed a nearly 1.9‐fold (~185%) larger size (Figure S4**D**) and exhibited ~1.5 times (~152%) more protrusions, compared to WT cells (Figure S4**E**).

### Lack of P1c in neurons leads to abnormal vesicle transport

**Figure 5:**
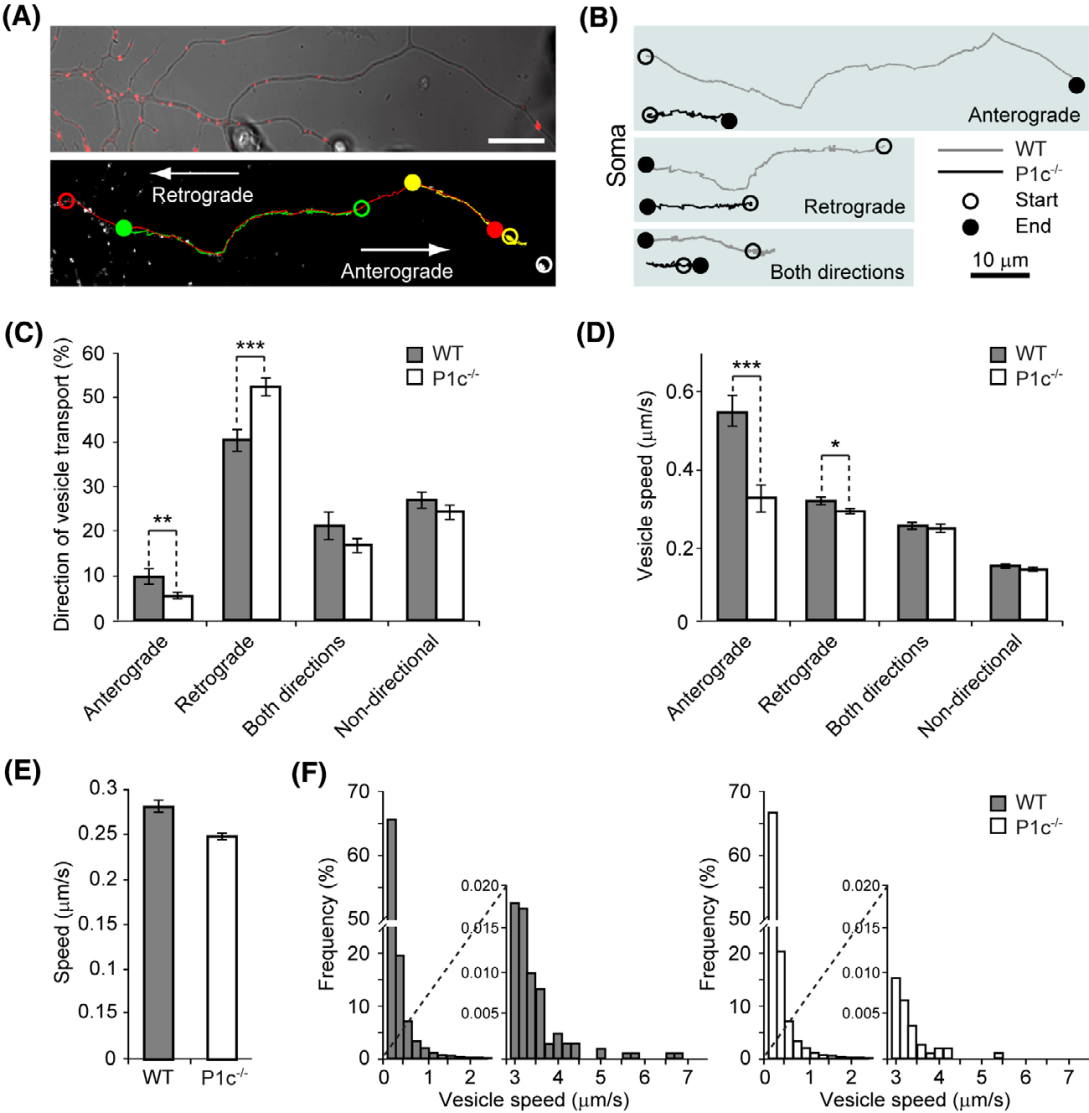
P1c‐deficiency affects axonal vesicle dynamics in dorsal root ganglion (DRG) neurons. (**A**) Micrograph of DRG neuronal processes under transmitted light with Lysotracker‐labelled vesicles (Ly‐vesicles) and the corresponding black and white micrograph showing the examples of vesicle trajectories in all analysed directions of vesicle movements along the axon (anterograde, retrograde, both directions, and non‐directional). Open circles denote start positions and full circles denote end positions of vesicle trajectories (red – anterograde, green – retrograde, yellow – both directions, white – non‐directional). Scale bar: 10 µm. (**B**) Schematic depictions of vesicle trajectories in WT and P1c^−/−^ mice. Note that vesicle trajectories are shorter in P1c^−/−^ mice, which is reflected also in speed measurements (panel **D**). (**C**) Directions of Ly‐vesicles undergoing anterograde, retrograde, bidirectional or non‐directional transport. The extent of vesicle movements is significantly lower in anterograde direction and significantly higher in retrograde direction in P1c^−/−^ mice compared to WT mice. Mean ± SEM [anterograde: WT (*n* = 169), P1c^−/−^ (*n* = 104); retrograde: WT (*n* = 645), P1c^−/−^ (*n* = 966); both directions: WT (*n* = 348), P1c^−/−^ (*n* = 314) non‐directional: WT (*n* = 477), P1c^−/−^ (*n* = 481)]. (**D**) Average speed of vesicles moving in different directions: anterograde or retrograde, or bidirectional, and non‐directional. Note that the average speed of vesicles moving in anterograde and retrograde directions is significantly lower in P1c^−/−^ compared to WT DRG neurons. Mean ± SEM [anterograde: WT (*n* = 169), P1c^−/−^ (*n* = 104); retrograde: WT (*n* = 645), P1c^−/−^ (*n* = 966); both directions: WT (*n* = 348), P1c^−/−^ (*n* = 314); non‐directional: WT (*n* = 477), P1c^−/−^ (*n* = 481)]. *P < 0.05, **P < 0.01, and ***P < 0.001 compared with WT; Mann–Whitney *U* test (**C**,**D**). (**E**) Average speed of vesicles (disregarding direction) in WT and in P1c^−/−^ mice. Mean ± SEM [WT (*n* = 1639), P1c^−/−^ (*n* = 1885)]. *P *= 0.341; Mann–Whitney *U* test. (**F**) Frequency distributions of vesicle speed within 2 s acquisition interval in DRG neuronal processes [WT (*n* = 171 802), P0 (*n* = 194 173 vesicles), four mice each]. The average speed of Ly‐vesicles (**D**) is significantly lower in P1c^−/−^ mice compared to WT mice mainly due to the lower number of vesicles with relatively high speed (>3 µm/s).

To assess the impact of P1c loss‐inflicted MT alterations on axonal vesicle transport, we treated WT and P1c^−/−^ DRG neurons with LysoTracker™ Red DND‐99, a red‐fluorescent dye used for labelling and tracking acidic organelles (Figure 5**A**) [20, 34, 35]. Monitoring anterograde, retrograde, and bi‐directional movement of vesicles in WT neurons, 11% of the vesicles were found to move anterogradely and 40% retrogradely, while in P1c^−/−^ neurons anterograde transport was down to 6% and retrograde transport up to 52%; the percentage of vesicles undergoing bidirectional or non‐directional movement remained unchanged (Figure 5**B**,**C**). Moreover, the average velocity of anterogradely moving vesicles was significantly lower in P1c^−/−^ (0.33 µm/s) compared to WT neurons (0.55 µm/s) and a significant decrease was also observed for retrogradely moving vesicles (0.29 µm/s in P1c^−/−^ *vs*. 0.32 µm/s in WT neurons) (Figure 5**D**). The overall average speed of dye‐labelled vesicles measured disregarding directionality was ~11% lower in DRG neurons of P1c^−/−^ compared to WT mice (Figure 5**E**). When we measured the frequency distributions of vesicle speed within 2 s acquisition intervals in neuronal processes, the majority of vesicle speed measurements were up to 0.2 µm/s, 65% in WT and 66% in P1c^−/−^ neurons (Figure 5**F**). Speed above 3 µm/s was detected in 0.07% of all measurements in WT and 0.03% in P1c^−/−^ neurons.

### Axonal translocation of mitochondria is differentially affected by P1c and P1b isoform deficiencies

**Figure 6:**
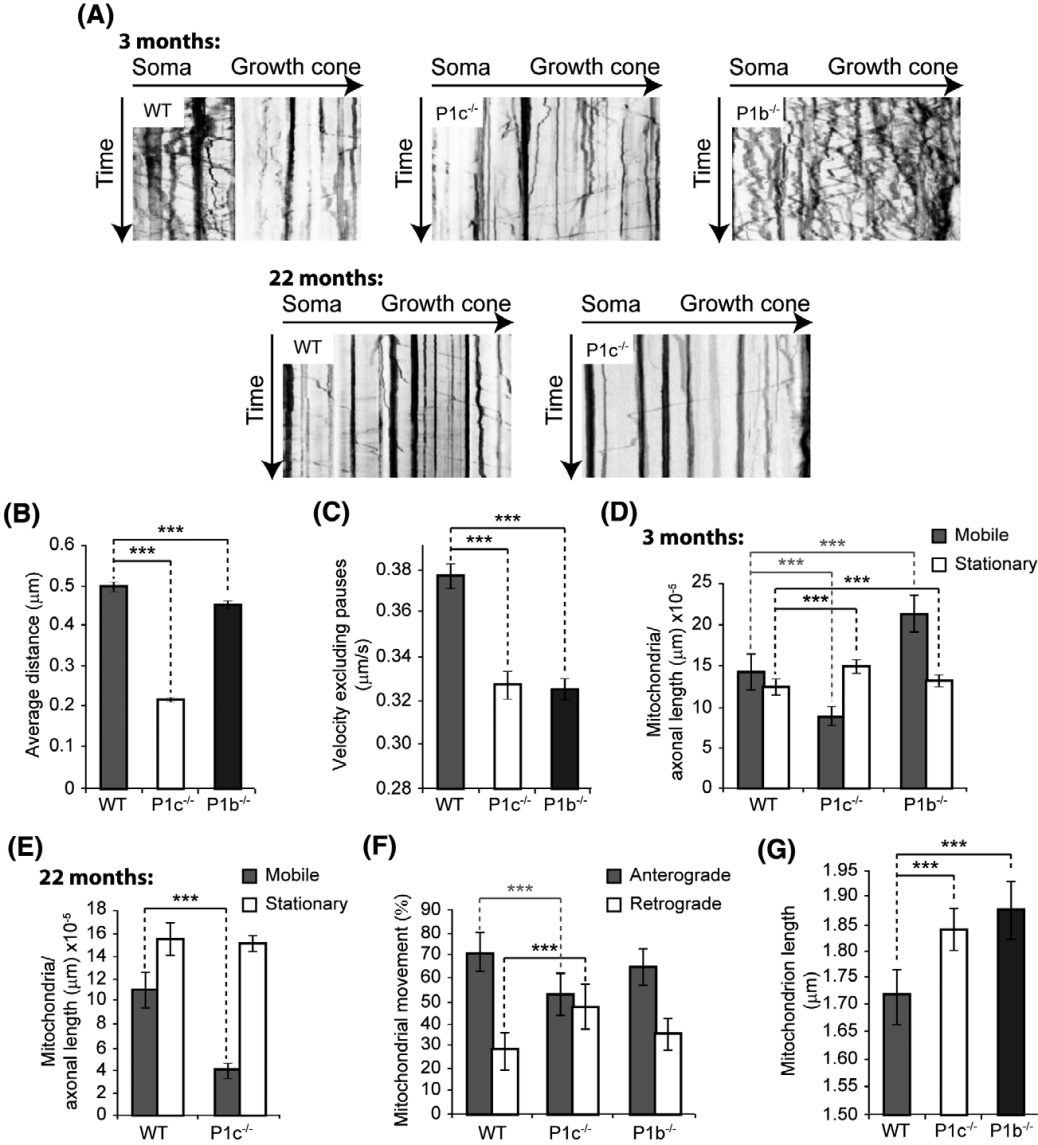
P1c‐deficiency impairs mitochondrial translocation in dorsal root ganglion (DRG) neurons. (**A**) Representative kymograph‐converted time‐lapse images (one frame every 2 s for 10 min) of MitoTracker‐labelled mitochondria in DRG neurons isolated from 3‐ and 22‐month‐old WT and P1c^−/−^, and 3‐month‐old P1b^−/−^ mice. Vertical and oblique tracks represent stationary and moving mitochondria, respectively. Note that the proportion of moving mitochondria (number of oblique tracks) is drastically reduced in 3‐month‐old P1c^−/−^ compared to WT neurons, while it is increased in corresponding P1b^−/−^ neurons. Also note, (i) increase in the proportion of non‐moving mitochondria in WT neurons with age (compare vertical tracks in 3 months *vs*. 22 months panels) and (ii) significant decrease in the total number of moving mitochondria in neurons from aged (22 months) P1c^−/−^ animals. (**B**,**C**) Analyses of the average distance covered and the average velocity of mitochondria (disregarding pauses), respectively, in neurons of 3‐month‐old WT and mutant mice. Mean ± SEM [WT (*n* = 180), P1c^−/−^ (*n* = 149), P1b^−/−^ (*n* = 244)]. Note reduction of both parameters in P1c^−/−^ as well as P1b^−/−^ neurons compared to WT controls. (**D**,**E**) Statistical evaluation of mobile and immobile mitochondria in DRG neurons of 3‐month‐old (**D**) and 22 month‐old (**E**) WT and mutant mice, normalized to axon lengths. Mean ± SEM [**D**: WT (*n* = 217), P1c^−/−^ (*n* = 427), P1b^−/−^ (*n* = 316); **E**: WT (*n* = 38), P1c^−/−^ (*n* = 87)]. (**F**,**G**) Directionality of mitochondrial translocation and length of individual mitochondria, respectively, in neurons of 3‐month‐old WT, P1c^−/−^ and P1b^−/−^ mice. Mean ± SEM [**F**: WT (*n* = 54), P1c^−/−^ (*n* = 93), P1b^−/−^ (*n* = 56); **G**: WT, mitochondria (*n* = 413), cells (*n* = 10); P1c^−/−^ (*n* = 708), (*n* = 10); P1b^−/−^ (*n* = 790), (*n* = 5)]. ***P < 0.001 compared with WT; unpaired Student’s *t* test (**B**–**G**).

It has been shown that tau pathology significantly inhibits mitochondrial transport, leading to degeneration of mitochondria and reduction of ATP production [36]. Since the proportion of MT‐bound tau was found to be increased in P1c‐deficient brain lysates as well as intact neurons isolated from P1c^−/−^ and P0 mice, we explored whether P1c deficiency also had an impact on mitochondrial translocation. As isoform P1b is the only isoform of plectin shown to specifically interact with mitochondria, thereby affecting respiratory functions [7, 37], P1b‐deficient neurons were included in the analysis to raise the prospects of gaining a better understanding of the underlying molecular mechanisms. To monitor the mobility of axonal mitochondria, we conducted time‐lapse imaging of WT, P1c^−/−^ and P1b^−/−^ DRG neurons that had been treated with MitoTracker, a mitochondrion‐selective dye specifically accumulating in respiring mitochondria. Based on kymographs of mobile mitochondria traced in neurons isolated from 3‐month‐old mice, the average distance travelled by mitochondria along axonal MT tracks was found to be drastically reduced (by ~56%) in P1c^−/−^ *vs*. WT neurons, while in P1b^−/−^ neurons it was decreased by only ~10% (Figure 6**A**, upper row; and Figure 6**B**). In either case, the average velocity of mobile mitochondria (pausing times excluded) was significantly decreased (from 0.38 µm/s in WT cells to 0.33 µm/s and 0.32 µm/s in P1c^−/−^ and P1b^−/−^ neurons, respectively) (Figure 6**C**). Interestingly, while in WT cells 53% of mitochondria were found to be mobile, in P1c^−/−^ cells the proportion of mobile mitochondria was reduced to 37%, but increased to 62% in P1b^−/−^ neurons (oblique kymograph tracks in Figure 6**A**, upper row; and Figure 6**D**). In senescent mice (22 months), the proportion of immobile axonal mitochondria compared to their 3 month‐old counterparts in WT mice showed an increase of 11 percentage points (from 47% to 58%), whereas for P1c^−/−^ neurons the corresponding increase amounted to 16 percentage points (from 63% to 79%) (compare vertical tracks in upper and lower row panels in Figure 6**A**; and bar graphs **D** and **E**). When the proportion of anterogradely (Figure 6**A**, oblique tracks starting from left hand side of kymographs) *vs*. retrogradely (oblique tracks starting from opposite site) moving mitochondria in neurons of all three genotypes was measured, in WT neurons 72% of motile mitochondria showed anterograde and 28% retrograde movement, whereas in P1c^−/−^ neurons only 52% of the mitochondria moved anterogradely (Figure 6**F**). With 65% anterogradely and 35% retrogradely moving organelles, P1b^−/−^ neurons resembled WT neurons (Figure 6**F**). Finally, mitochondria in P1c^−/−^ and P1b^−/−^ DRG neurons were found to be significantly longer compared to WT cells (Figure 6**G**), reminiscent of a similar phenotype observed in P1b‐deficient fibroblasts and myocytes [7, 37].

### Decreased pain sensitivity, impaired fear‐conditioned memory and altered long‐term memory of P1c‐deficient mice

**Figure 7:**
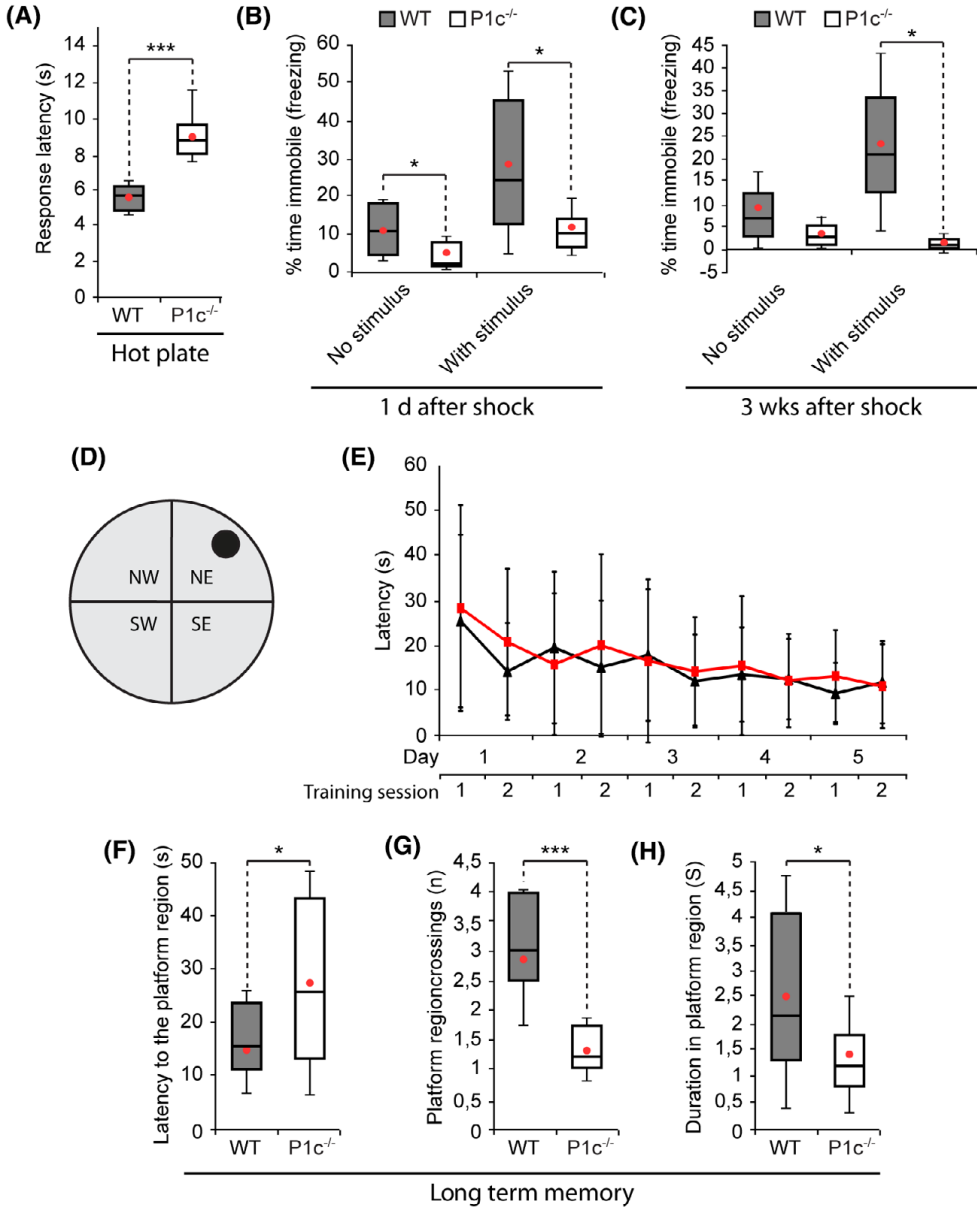
Behavioural assays performed on P1c^−/−^ *vs*. WT mice. (**A**) Box and whisker plot of pain response of WT and P1c^−/−^ mice assessed as the time between the placement of the mice on a hot metal plate and their first pain‐related reaction. The rectangle represents the interquartile range for first to third quartile and the horizontal line within the box is the median. The red dot within the box marks the mean. Whiskers above and below the box represent the mean ± SD. Mice: WT (*n* = 6), P1c^−/−^ (*n* = 6). (**B**,**C**) Box and whisker plots of fear conditioned memory of WT and P1c^−/−^ mice. Animals were initially trained by repeated exposure to a mild foot shock co‐terminated with an auditory cue. For evaluation of fear‐conditioned short‐ and long‐term memory, mice were positioned in an altered chamber and were exposed to the auditory cue 1 day (**B**) or 3 weeks (**C**), respectively, after the training phase. Plot characteristics as in (**A**). Note impaired fear‐conditioned memory of P1c^−/−^ compared to WT mice. Mice: WT (*n* = 8), P1c^−/−^ (*n* = 6). *P < 0.05 and ***P < 0.001 compared with WT, Mann–Whitney *U* test (**A**–**C**). (**D**–**H**) Morris water maze test. (**D**) Scheme of pool where the animals had to find a platform (black circle) hidden beneath the water surface, using visual cues placed on the walls. NW, NE, SE and SW, pool quadrants. (**E**) WT (black line) and P1c^−/−^ (red line) mice were trained for 5 days (twice per day) and latencies of finding the platform were recorded. WT and P1c^−/−^ mice did not show any significant differences during the learning process. Mean ± SD [mice: WT (*n* = 8), P1c^−/−^ (*n* = 6)]. (**F**–**H**) Box and whisker plots of long‐term memory assessment by recording latencies to reach the platform region (**F**), number of crossings over the platform region (**G**), and overall continuance in the region (**H**), 76 h after the training phase. Plot characteristics as in (**A**). Mice: WT (*n* = 8), P1c^−/−^ (*n* = 6); *P < 0.05 and ***P < 0.001 compared with WT; Mann–Whitney *U* test. Note, P1c^−/−^ mice showed impaired long‐term memory compared to WT littermates by the criteria of the trial.

As the primary sensory neurons of the peripheral nervous system, DRG neurons are responsible for the generation and propagation of pain when excited with thermal, mechanical or chemical stimuli at noxious range [38]. To test whether the phenotypic alterations observed *ex vivo* in P1c^‐/‐^ DRG neurons were physiologically relevant, P1c^−/−^ and WT mice were subjected to a standard nociceptive test, in which heat hyperalgesia was assessed by measuring escape latencies from a thermally‐controlled metal plate of 52–55°C. As shown in Figure 7**A** P1c^−/−^ mice reacted to the stimulus on average only after 9 s, while their WT counterparts showed a first reaction already after 5.5 s, confirming that the phenotypes observed ex vivo were having functional consequences in the context of the whole organism.

Representing part of the brain system that is responsible for spatial memory, hippocampal neurons showed phenotypes similar to those of sensory DRG neurons. Hence, we used P1c^−/−^ mice also as a model to investigate a possible role of plectin in learning and memory. First, we applied fear conditioning to either a cue or context, which represents a form of associative learning and memory test for aversive events and environmental cues, involving the amygdala and, particularly for context conditioning, the hippocampus [39]. When contextual learning was measured by monitoring the freezing response of the animal 1 day and 3 weeks after fear conditioning, P1c^−/−^ mice showed no significant alterations in context recognition compared to WT mice (data not shown). However, in a similar evaluation of cue conditioning, P1c^−/−^ mice exhibited significantly reduced fear conditioned memory to an auditory cue compared to their WT counterparts after 1 day of training (Figure 7**D**), and still performed worse than their WT counterparts when tested again 3 weeks later (Figure 7**E**). Since WT and P1c^−/−^ mice showed similar reactions immediately after mild foot shock applications (data not shown), it is unlikely that differences in their pain sensitivity (see Figure 7**A**) were playing a role in this type of memory tests.

In addition, we employed the Morris water maze spatial navigation task for assessing learning and memory abilities of rodents [22], where the animals had to swim to find a hidden platform using visual cues placed on the walls of a pool in order to escape from the aversive environment of water by the quickest and most direct route. During the acquisition phase, where the mice were placed in any of the four pool quadrants (Figure 7**F**) and allowed to find the platform, P1c^−/−^ mice performed comparably to their WT counterparts, as judged from the latency in finding the hidden platform (Figure 7**G**). When the platform was removed immediately after the acquisition phase, and the animals were monitored for their latency in reaching the platform area, frequency of crossing it, and duration of staying within its boundaries, no significant differences between WT and P1c^−/−^ mice were revealed, indicating similar short‐term memory functioning (data not shown). However, when subjected to similar trials 72 h later, P1c^−/−^ mice presented with a strikingly increased searching time, crossed the platform region less frequently, and remained less time within its boundaries, compared to their WT counterparts (Figure 7**F**–**H**). These symptoms suggested that P1c^−/−^ mice suffered from impaired long‐term memory. In conclusion, lack of P1c in neurons exerts functional deficits, such as impaired pain sensitivity, reduced fear conditioned memory, and impaired long‐term memory.

## Discussion

We show here that deficiency in P1c, the plectin isoform most prominently expressed in neural tissues and cells, leads to increased tau protein association and altered dynamics of neuronal MTs, with consequences for neuritogenesis and organelle trafficking. In addition, we found that P1c deficiency affects pain sensitivity and fundamental brain functions such as learning and memory of mice. Our findings have implications not only for plectinopathies involving the nervous system but also neuropathies associated with dysfunctional tau protein.

Our results show that P1c specifically associates with MTs along the shafts as well as in the growth cones of DRG and hippocampal neurons. In fact, co‐sedimentation assays showed that the P1c‐specific N‐terminal fragment, in combination with the plectin ABD version containing short exon 2α‐ and 3α‐encoded sequence insertions (P1c(2α3α)‐8), formed an optimal binding domain for MTs assembled from MAP‐free tubulin. However, binding of P1c to MTs was not restricted to isoforms with ABDs containing exon 2α‐ and 3α‐specific sequences, because N‐terminal fragments of P1c without 2α‐ and 3α insertions (P1c‐8) showed binding as well, though at reduced levels. Likewise, a direct interaction of P1c‐8 with MTs has been shown also for keratinocytes, a cell type that expresses exclusively P1c transcripts without 2α/3α‐specific sequences [23]. Hence, without constituting a strict requirement, the combination of exon1c with exons 2α and 3α appears to present an ideal alliance in forming an optimal MT‐binding interface, a molecular feature that probably is of particular importance for neural tissues.

Cell fractionation as well as co‐localization assays revealed elevated levels of tau proteins bound to MTs of DRG and hippocampal neurons in the absence of P1c. As normal levels could be restored in P1c^−/−^ cells by forced expression of full‐length P1c‐GFP fusion proteins, P1c deficiency seemed directly linked to and causative for elevated tau association with MTs. Consistent with a previous study on the role of P1c in keratinocytes [23], we found that plectin’s SH3 domain was crucial for tau‐binding. These observations support a mechanistic model where P1c is targeted directly to the surface of axonal MTs via its isoform‐specific N‐terminal sequence domain, whereas its further downstream residing SH3 domain binds to and impairs the MT‐binding capacity of tau. Plectin‐MAP interactions ought not to be restricted to isoform P1c, as all known isoforms of plectin express an SH3 domain. A close enough localization of IFs and MTs, e.g. at crossover sites or upon longitudinal alignment, might be sufficient for plectin‐MAP interaction to occur, as has been suggested for MT networks in skeletal muscle fibres [40]. Moreover, as SH3 domains are found in many proteins, including regulators of MAPs such as the kinases Fyn and Src [29, 41], proteins other than plectin may exert similar functions. The mechanism of plectin‐tau interaction and the location of tau's plectin‐binding domain(s) are presently unknown. Considering that tau belongs to the group of intrinsically disordered proteins which lack a unique structure and exist in multiple, quickly interconverting conformations [42], it will be a challenging task for future research to unravel these mechanisms and also determine whether different isoforms of tau show differential plectin‐binding capacities.

Our results are in line with a recent study of Qiang *et al*. showing that MAP6, a genuine MT stabilizer, is enriched along the stable domains of MTs, while tau is associated with their more labile domains [26]. Thus, being enriched on the labile domains of MTs, tau was suggested not to act as a stabilizer of axonal MTs, as presumed in many previous studies, but rather as a provider of longer labile domains, thereby promoting MT assembly and concomitantly limiting the binding of stabilizers such as MAP6 [26]. In this context, it is of interest to note that with respect to MTs, P1c deficiency phenotypically manifested similarly to MAP6 depletion, but conversely to tau depletion [26]. The observation of increased posttranslational acetylation of α‐tubulin in P1‐deficient neurons would be consistent with the notion that the MTs in stable axonal areas of these cells were more accessible to this modification than those of WT cells. As phenotypic features of P1c^−/−^ cells, such as increased acetylation of tubulin, could be restored by forced expression of full‐length P1c, but not truncated protein versions lacking the rod and C‐terminal IF‐binding domains, our data imply that P1c required association with neurofilaments in order to fulfil its function.

The most evident morphological alterations of P1c‐deficient DRG and hippocampal neurons were the reduction in axonal branching and the increase in growth cone sizes. A number of studies are consistent with these observations. Regarding branching, Yu *et al*. found that the presence of tau on MTs shielded them against the action of the MT severing protein katanin; accordingly, axons depleted of tau showed a greater propensity for branching [43]. Along these lines, one could expect axons with elevated levels of MT‐bound tau, such as those of P1c^‐/‐^ neurons, to exhibit decreased levels of branching. Moreover, MT stabilization achieved by low doses of taxol has been reported to induce axon formation [44] and to promote the extension of newly polymerized MTs to the distal part of the neurite, including the growth cone. Thus, the hyper‐stabilized MT domains hallmarking P1c^−/−^ neurons are likely to act as nucleation seeds for more actively assembling and protruding MTs, resulting in increased neurite length and more spread, i.e. larger growth cones.

Assessing axonal organelle trafficking, the average speed and the mainly anterograde directionality of mitochondrial movements observed in WT neurons was in agreement with a previous study on mouse DRG neurons [34]. The observation that Lysotracker‐labelled vesicles, contrary to mitochondria, were predominantly engaged in retrograde movements was also consistent with this previous report, as well as with a report by Miyake *et al*., who monitored identically labelled vesicles in retinal ganglion cells [35]. Furthermore, the observed average speeds of Lysotracker‐labelled vesicles and the ratio of retrograde *vs*. anterograde movements were similar to those reported [34]. Interestingly, for both, axonal Lysotracker‐labelled vesicles and mitochondria, we observed reduced transport rates, a general reduction of mobile species, and a general shift from anterograde (kinesin‐mediated) towards retrograde (dynein‐mediated) movements in P1c^−/−^ neurons. The most plausible mechanistic explanation for the observed phenotypes was the elevated levels of MT‐bound tau protein found in mutant cells. This notion is consistent with reports showing that (i) tau pathology inhibits mitochondrial transport and leads to degeneration and reduced ATP production of mitochondria [36], and (ii) overexpression of tau protein in N2a cells leads to diminished transport of Golgi‐derived vesicles into axons [25].

Among the key components of the machinery accomplishing active transport along MTs are the molecular motor proteins dynein and kinesin, which transport cargo towards the MT minus (cell body) or plus (cell periphery) ends, respectively [24]. In the axon, dynein and kinesin compete with non‐motile MAPs, which, being directly bound to MTs, block the path of motor proteins. It has been shown that tau inhibits kinesin activity *in vivo* and *in vitro* [45], and studying single molecules of motor proteins moving along tau‐decorated MTs, Dixit *et al*. reported that kinesin detached at patches of MT‐bound tau, whereas dynein needed 10 times higher tau levels to be affected [24]. These studies indicated that MAPs can spatially regulate the balance of MT‐dependent axonal transport. Thus, increased levels of tau, as observed in the case of P1c deficiency, tend to inhibit kinesin‐mediated anterograde transport. It follows that P1c, by reducing the levels of MT‐bound tau, provides a mechanism for spatiotemporal regulation of axonal transport.

Measuring mitochondrial mobility parameters in P1c‐ and P1b‐deficient DRG neurons in parallel enabled us to gain new insights into the differential impacts of distinct plectin isoforms on these organelles. Deficiency in P1b, the only isoform of plectin that specifically interacts and interlinks mitochondria with the cytosolic IF network, has been shown to lead to alterations in morphology (elongation) and homeostasis of mitochondrial networks in fibroblasts, myoblasts, and myoblast‐derived differentiated myotubes [7, 37]. In neurons, we observed similar alterations of mitochondrial network morphology not only in the absence of P1b but also of P1c. However, a number of other phenotypic features, such as drastically reduced average distances travelled by mitochondria along axonal MT tracks, an overall lesser mobility of mitochondria that increased with age, as well as a shift from anterograde towards more retrograde transport, were observed only in P1c‐, but not P1b‐deficient neurons. On the other hand, significantly increased levels of mobile mitochondria were found only in P1b‐deficient neurons. Based on these data, we propose that P1b, as a direct bridging element between mitochondria and the IF network, is involved in subcellular positioning and immobilization of mitochondria, while P1c is responsible for MT‐mediated mitochondrial transport. Accordingly, a lack of P1b makes mitochondria more mobile (due to lesser anchorage), while a lack in P1c results in a shift from anterograde towards retrograde movement. Interference in either pathway effects morphological alterations, resulting in increased mitochondrial length. Whether the impairment of MT‐dependent mitochondrial transport due to P1c deficiency also results in functional deficits of the organelle, remains to be investigated.

Finally, our study demonstrates that the phenotypic alterations observed *ex vivo* in P1c‐deficient DRG and hippocampal neurons were physiologically significant, as mice lacking specifically this isoform showed functional deficits, such as impaired pain sensitivity, reduced fear conditioned memory, and impaired long‐term memory. In agreement with our phenotypic *ex vivo* and *in vivo* analyses, numerous studies point towards a role of MT dynamics in learning and memory. For instance negative regulation of MT formation by the phosphoprotein stathmin was found to be critical for memory and to be disrupted in aging [46]. Another example are rats that exhibited memory impairment and performed significantly worse in the Morris water maze test when treated with colchicine, a MT‐depolymerizing drug leading to the disruption of axonal transport [47]. Interestingly, similar to P1c^−/−^ mice, tau knockout mice showed impaired contextual and cued fear conditioning [48]. Furthermore, transgenic mice, expressing a mutated form of tau (tau_P301L_) linked to hereditary tauopathy, performed in the Morris water maze test significantly worse than normal animals, while suppression of the mutant protein improved memory functions [49]. Moreover, Barten *et al*. could demonstrate that the hyperdynamic state of MTs and the cognitive defects of tau transgenic mice expressing abnormal forms of tau, could be improved by treating the animals with the MT‐stabilizing agent BMS‐241027 [50]. The notion arising from these studies, namely that cognitive functions based on learning and memory are dependent on tightly controlled MT dynamics involving tau protein, is supported by the here reported phenotypes of P1c‐deficient mice, including hyperdynamic (destabilized) axonal MTs combined with increased tau‐MT association and cognitive dysfunctions.

The defects in axonal transport processes are associated with neurodevelopmental or neurodegenerative disorders, including Alzheimer’s disease or Huntington’s disease [51]. Likewise, hyperphosphorylation of tau in Alzheimer’s disease and frontotemporal dementia results in reduced binding ability of tau to MTs and impairment of axonal transport [52, 53]. Accordingly, the here established new role of plectin in modulating neuronal functions is expected to have clinical implications. Indeed, signs of neurodegenerative disorders including brain atrophy have been reported for a couple of EBS‐MD patients [4, 5]. Moreover several patients suffering from EBS‐MD‐MyS, where neuromuscular transmission is impaired, have been described [54, 55], which is consistent with the loss of neuromuscular synapse integrity upon plectin knockout in skeletal muscle of mice [56]. Finally, there have been first reports of isoform‐specific human plectinopathies caused by homozygous mutations in the first exons of P1f and P1a, leading to autosomal‐recessive LGMD2Q [57] and skin‐only EBS without extracutaneous involvement [58], respectively. Thus, regarding undiagnosed neuropathies and neurological disorders, additional, and potentially isoform P1c‐specific, plectin mutations can be expected to be identified in the near future. In this context, the analysis of mutations in P1c‐specific sequences should become standard in clinical diagnostic screening programs.

In summary, our findings not only open up new insights into the impact of neuronal cytoskeleton organization on fundamental functions of sensory as well as hippocampal neurons, but also provide a basis towards the development of strategies for the treatment of neurological disorders associated with plectin and tau protein.

## Author contributions

RGV, EM and LW, contributed to conception and design, acquisition of data, analysis and interpretation of data, drafting or revising the article. KB, IF, GWa, JJ, MP and RZ contributed to acquisition of data, analysis and interpretation of data. GWi contributed to conception and design, analysis and interpretation of data, drafting and revising the article.

## Ethical approval

All procedures performed in the study were in accordance with the ethical standards of the University of Vienna.

## Supporting information


**Figure S1.** Plectin‐dependent association of microtubule‐associated proteins (MAPs) with neuronal MTs assessed by co‐fractionation.
**Figure S2.** Relative tau/tubulin signal intensities measured in hippocampal neurons of WT and P0 mice.
**Figure S3.** Acetylation state of axonal MTs in hippocampal neurons of WT and P0 mice.
**Figure S4.** P1c‐deficiency affects neurite branching and growth cone morphology in hippocampal neurons of WT and P0 mice.
**Table S1.** Primary antibodies used in this study.
**Video Clip S1.** Time‐lapse images of EB3‐mCherry comets in a WT DRG neuron.
**Video Clip S2.** Time‐lapse images of EB3‐mCherry comets in a P1c^−/−^ DRG neuron.
**Video Clip S3.** Time‐lapse images of vesicles in WT DRG neurons.
**Video Clip S4.** Time‐lapse images of vesicles in P1c^−/−^ DRG neurons.
**Video Clip S5.** Time‐lapse images visualizing MitoTracker‐labelled mitochondria in DRG neurons isolated from 3 month‐old WT mice.
**Video Clip S6.** Time‐lapse images visualizing MitoTracker‐labelled mitochondria in DRG neurons isolated from 3 month‐old P1c^−/−^ mice.
**Video Clip S7.** Time‐lapse images visualizing MitoTracker‐labelled mitochondria in DRG neurons isolated from 3 month‐old P1b^−/−^ mice.

## Data Availability

Data are available on request from the authors.
