# Supplementary material for: Plectin dysfunction in neurons leads to tau accumulation on microtubules affecting neuritogenesis, organelle trafficking, pain sensitivity and memory
Source: Neuropathol Appl Neurobiol. 2020 Jun 25;47(1):73–95. doi: 10.1111/nan.12635 (PMC7891324; doi:10.1111/nan.12635)
Supplement: Supplementary file 1 — Figure S1. Plectin‐dependent association of microtubule‐associated proteins (MAPs) with neuronal MTs assessed by co‐fractionation. Figure S2. Relative tau/tubulin signal intensities measured in hippocampal neurons of WT and P0 mice. Figure S3. Acetylation state of axonal MTs in hippocampal neurons of WT and P0 mice. Figure S4. P1c‐deficiency affects neurite branching and growth cone morphology in hippocampal neurons of WT and P0 mice. Table S1. Primary antibodies used in this study. Video Clip S1. Time‐lapse images of EB3‐mCherry comets in a WT DRG neuron. Video Clip S2. Time‐lapse images of EB3‐mCherry comets in a P1c−/− DRG neuron. Video Clip S3. Time‐lapse images of vesicles in WT DRG neurons. Video Clip S4. Time‐lapse images of vesicles in P1c−/− DRG neurons. Video Clip S5. Time‐lapse images visualizing MitoTracker‐labelled mitochondria in DRG neurons isolated from 3 month‐old WT mice. Video Clip S6. Time‐lapse images visualizing MitoTracker‐labelled mitochondria in DRG neurons isolated from 3 month‐old P1c−/− mice. Video Clip S7. Time‐lapse images visualizing MitoTracker‐labelled mitochondria in DRG neurons isolated from 3 month‐old P1b−/− mice. [file NAN-47-73-s001.zip › nan12635-sup-0013-Supinfo.docx]

**Plectin dysfunction in neurons leads to tau accumulation on microtubules affecting neuritogenesis, organelle trafficking, pain sensitivity and memory**

**Supporting information**

**Supplemental figure legends**

**Figure S1.** Plectin-dependent association of microtubule-associated proteins (MAPs) with neuronal MTs assessed by co-fractionation (A) Cell lysates prepared under MT-stabilizing conditions from WT and P1c-deficient mouse brain were fractionated, and total cell lysates, MT-unbound, and MT-bound fractions were analysed by immunoblotting using antibodies as indicated. (B-E) MAP1A/1B, MAP2, MAP1A light chain (LC) and MAP1B LC signal intensities in MT-bound fractions, as shown in (A), normalized to tubulin levels. Note, while increased levels of tau were found in P1c^-/-^ compared to WT samples (see Figures 2A and 2B), the levels of MAP1A and MAP1B LCs were decreased in P1c^-/-^ samples, while the levels of MAP1A/1B and MAP2 remained unchanged. Mean ± SEM (n=3); ***p*<0.01 and ****p*<0.001 compared with WT; unpaired Student’s *t* test..

**Figure S2.** Relative tau/tubulin signal intensities measured in hippocampal neurons of WT and P0 mice. (A) Neurons isolated from hippocampi of WT and P0 mice were subjected to IFM after double-labelling for tau and tubulin. Scale bar: 20 µm. (B) Statistical evaluation of tau-specific signal intensities (normalized to tubulin levels) measured in untransfected WT and P0, and full-length P1c-transfected P0 hippocampal neurons (all signals were recorded below saturation levels). Mean ± SEM [axons analysed: WT (n=25), P0 (n=14), P0 + P1c(2α3α)-32 (n=23)]; ****p*<0.001 compared with WT; one-way ANOVA and post-hoc Tukey correction. Note, i) 1.7-fold higher tau to tubulin signal ratio in P0 cells, and ii) transfection of P0 cells with full-length P1c restored their tau/tubulin signal level to below that of WT controls.

**Figure S3.** Acetylation state of axonal MTs in hippocampal neurons of WT and P0 mice. (A) IFM of WT and P0 hippocampal neurons using antibodies to acetylated and total tubulin. Scale bar: 20 µm. (B) Statistical evaluation of acetylated to total tubulin signal intensity ratios in neurons as shown in (A). Mean ± SEM [WT (n=35), P0 (n=34)]; ****p*<0.001 compared with WT; unpaired Student’s *t* test.

**Figure S4.** P1c-deficiency affects neurite branching and growth cone morphology in hippocampal neurons of WT and P0 mice. (A) Classification of hippocampal neurons according to the number of axonal branching points. Low, medium, and high branching was assigned to neurons with <2.0, 2.0-4.0, and >4.0 branching points per neurite length (measured in pixels ×10^2^), respectively. Scale bar: 20 µm. (B) Statistical analysis of branching in neurons isolated from WT and P0 mice [WT (n=34), P0 (n=28)]. (C) Growth cones of WT and P0 hippocampal neurons were visualized by labelling of filamentous actin with fluorescent phalloidin. Scale bars: 5 µm. (D,E) Statistical evaluation of growth cone areas (D) and perimeters (E). Mean ± SEM (n=30 each); ****p*<0.001 compared with WT; unpaired Student’s *t* test.

**Supplemental tables**

**Table S1.** Primary antibodies used in this study.

**Supplemental video legends**

**Video S1.** Time-lapse images of EB3-mCherry comets in a WT DRG neuron. Images were collected using a spinning-disk microscope (for details see text) at 2 sec intervals during a period of >5 min. Arrowheads, EB3 comets (corresponding to growing tips of MTs) moving in a growth cone. Note that MTs approach the cell membrane (cyan arrowhead, time intervals 164-382 sec), pause (cyan arrowhead, time point 440 sec) and undergo catastrophe (cyan arrowhead, 441-552 sec). Yellow arrowhead points at another MT that undergoes catastrophe upon reaching the cell periphery (time point 10 sec).

**Video S2.** Time-lapse images of EB3-mCherry comets in a P1c^-/-^ DRG neuron. Images were collected as described for Video S1. Arrowheads, EB3 comets moving in a growth cone. Note higher number of EB3-mCherry comets per growth cone area in P1c^-/-^ DRG neurons compared to WT cells (compare with Video S1). Also note that MTs in P1c^-/-^ DRG neurons upon reaching the cell margins (yellow arrowhead, time point 10 sec), start bending (yellow arrowhead, time interval 11-44 sec) and continue growing (yellow arrowhead, time interval 44-120 sec). Cyan and violet arrowheads, other MTs that continue growing upon reaching the cell periphery (time interval 126-256 sec and 296-396 sec, respectively).

**Video S3**. Time-lapse images of vesicles in WT DRG neurons. Synaptic vesicles were labelled with LysoTracker™ Red DND-99 dye and images were collected using a confocal microscope for 240 sec. To make the video more demonstrative, its speed was tenfold increased.

**Video S4**. Time-lapse images of vesicles in P1c^-/-^ DRG neurons. Synaptic vesicles were visualized and images collected as described for Video S3.

**Video S5.** Time-lapse images visualizing MitoTracker-labelled mitochon­dria in DRG neurons isolated from 3 month-old WT mice. Images were collected using a spinning-disk microscope (for details see text) at 2 sec intervals during a period of 10 min.

**Video S6.** Time-lapse images visualizing MitoTracker-labelled mitochondria in DRG neurons isolated from 3 month-old P1c^-/-^ mice. Images were collected as described for Video S5.

**Video S7.** Time-lapse images visualizing MitoTracker-labelled mitochondria in DRG neurons isolated from 3 month-old P1b^-/-^ mice. Images were collected as described for Video S5.
