# Supplementary figures and images for: Plectin dysfunction in neurons leads to tau accumulation on microtubules affecting neuritogenesis, organelle trafficking, pain sensitivity and memory
Source: Neuropathol Appl Neurobiol. 2020 Jun 25;47(1):73–95. doi: 10.1111/nan.12635 (PMC7891324; doi:10.1111/nan.12635)

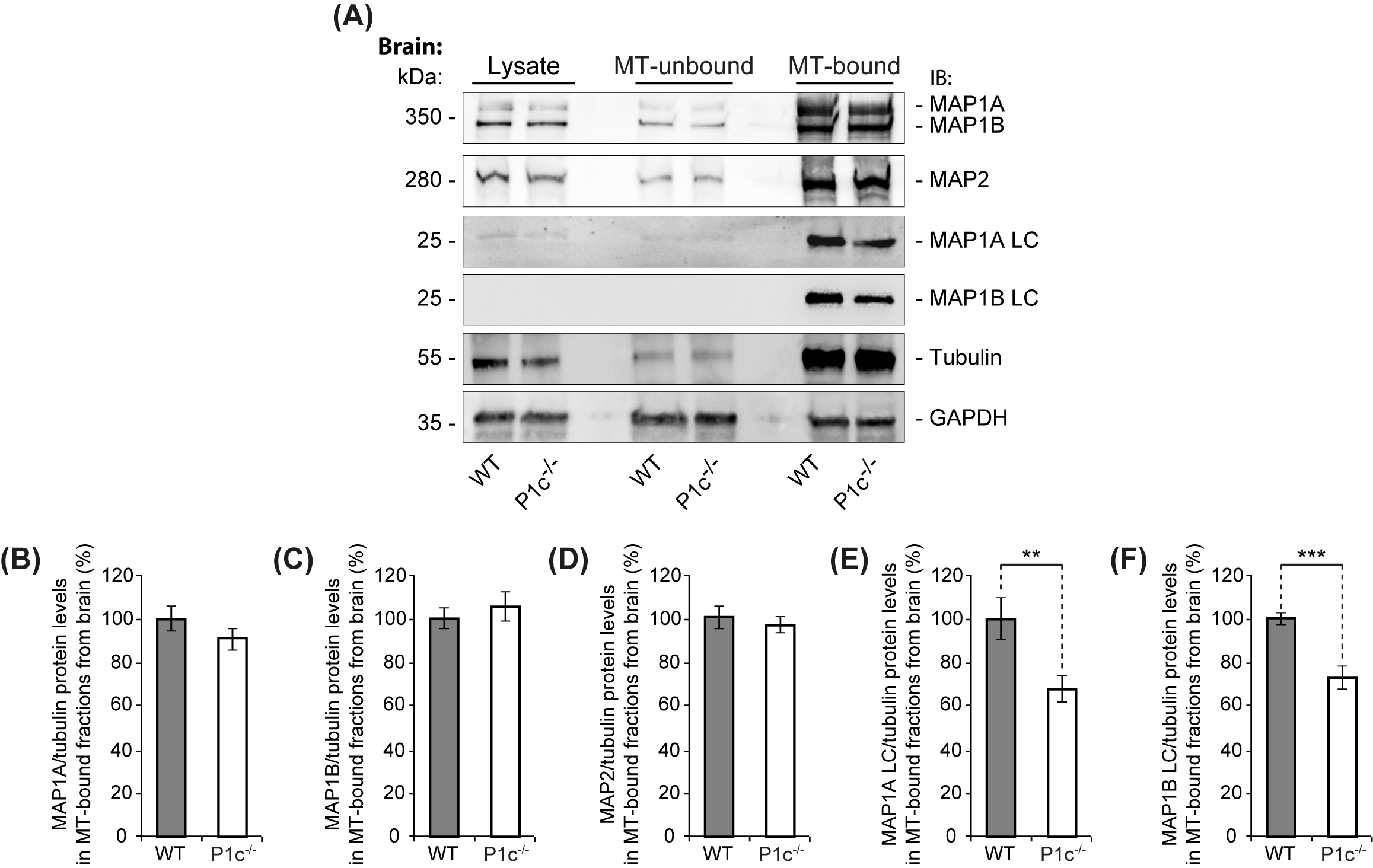

Supplement: Supplementary file 1 — Figure S1. Plectin‐dependent association of microtubule‐associated proteins (MAPs) with neuronal MTs assessed by co‐fractionation. Figure S2. Relative tau/tubulin signal intensities measured in hippocampal neurons of WT and P0 mice. Figure S3. Acetylation state of axonal MTs in hippocampal neurons of WT and P0 mice. Figure S4. P1c‐deficiency affects neurite branching and growth cone morphology in hippocampal neurons of WT and P0 mice. Table S1. Primary antibodies used in this study. Video Clip S1. Time‐lapse images of EB3‐mCherry comets in a WT DRG neuron. Video Clip S2. Time‐lapse images of EB3‐mCherry comets in a P1c−/− DRG neuron. Video Clip S3. Time‐lapse images of vesicles in WT DRG neurons. Video Clip S4. Time‐lapse images of vesicles in P1c−/− DRG neurons. Video Clip S5. Time‐lapse images visualizing MitoTracker‐labelled mitochondria in DRG neurons isolated from 3 month‐old WT mice. Video Clip S6. Time‐lapse images visualizing MitoTracker‐labelled mitochondria in DRG neurons isolated from 3 month‐old P1c−/− mice. Video Clip S7. Time‐lapse images visualizing MitoTracker‐labelled mitochondria in DRG neurons isolated from 3 month‐old P1b−/− mice. [file NAN-47-73-s001.zip › nan12635-sup-0001-FigureS1.tif]

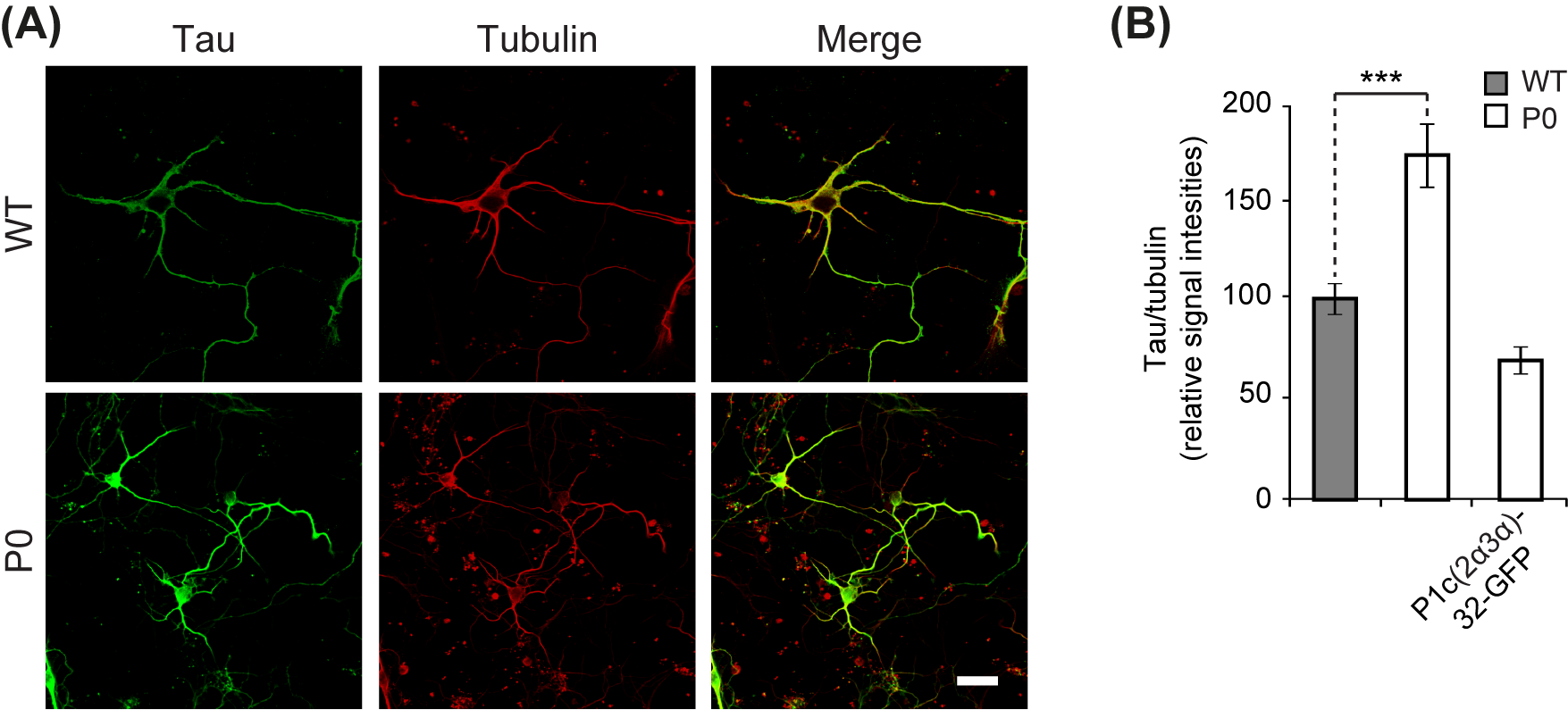

Supplement: Supplementary file 1 — Figure S1. Plectin‐dependent association of microtubule‐associated proteins (MAPs) with neuronal MTs assessed by co‐fractionation. Figure S2. Relative tau/tubulin signal intensities measured in hippocampal neurons of WT and P0 mice. Figure S3. Acetylation state of axonal MTs in hippocampal neurons of WT and P0 mice. Figure S4. P1c‐deficiency affects neurite branching and growth cone morphology in hippocampal neurons of WT and P0 mice. Table S1. Primary antibodies used in this study. Video Clip S1. Time‐lapse images of EB3‐mCherry comets in a WT DRG neuron. Video Clip S2. Time‐lapse images of EB3‐mCherry comets in a P1c−/− DRG neuron. Video Clip S3. Time‐lapse images of vesicles in WT DRG neurons. Video Clip S4. Time‐lapse images of vesicles in P1c−/− DRG neurons. Video Clip S5. Time‐lapse images visualizing MitoTracker‐labelled mitochondria in DRG neurons isolated from 3 month‐old WT mice. Video Clip S6. Time‐lapse images visualizing MitoTracker‐labelled mitochondria in DRG neurons isolated from 3 month‐old P1c−/− mice. Video Clip S7. Time‐lapse images visualizing MitoTracker‐labelled mitochondria in DRG neurons isolated from 3 month‐old P1b−/− mice. [file NAN-47-73-s001.zip › nan12635-sup-0002-FigureS2.tif]

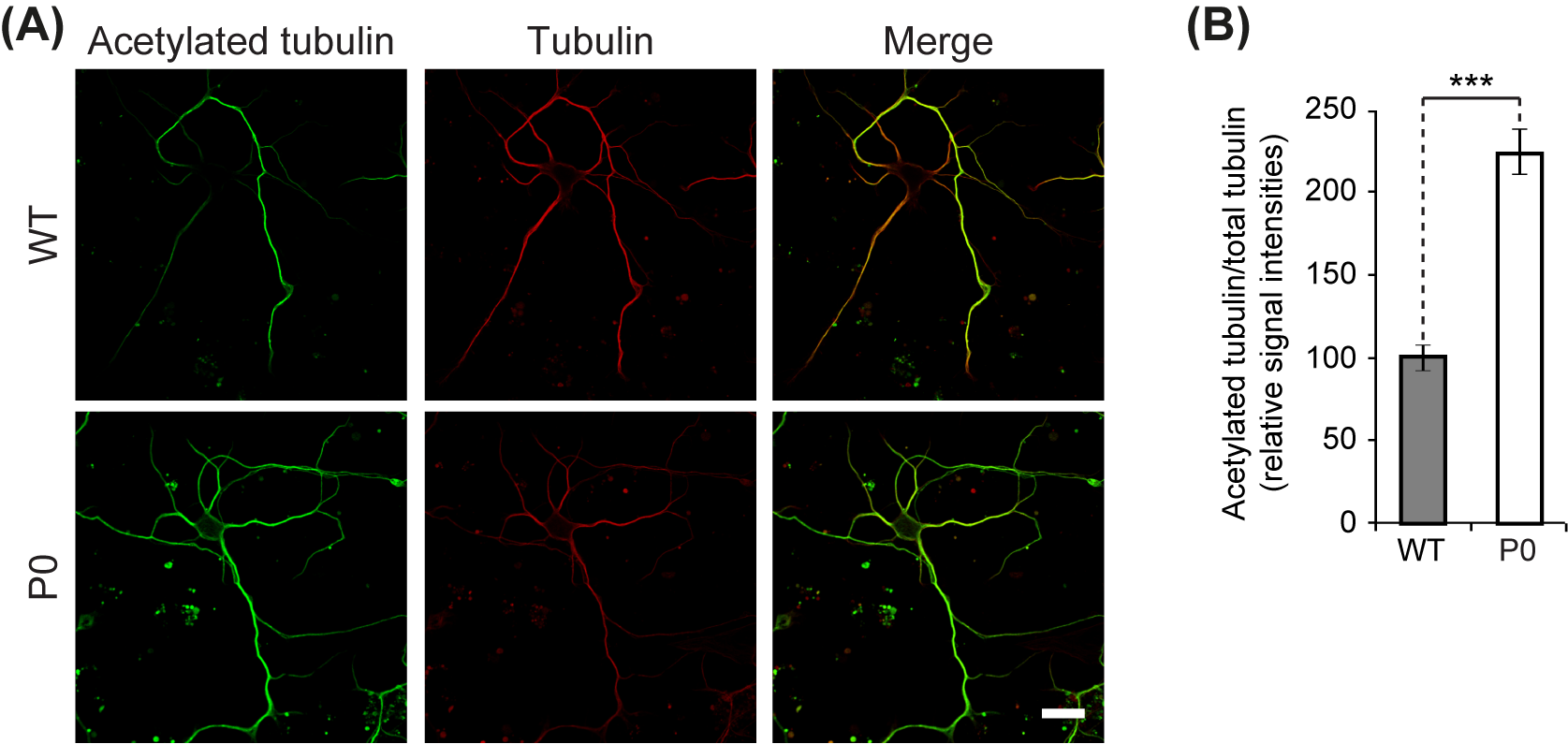

Supplement: Supplementary file 1 — Figure S1. Plectin‐dependent association of microtubule‐associated proteins (MAPs) with neuronal MTs assessed by co‐fractionation. Figure S2. Relative tau/tubulin signal intensities measured in hippocampal neurons of WT and P0 mice. Figure S3. Acetylation state of axonal MTs in hippocampal neurons of WT and P0 mice. Figure S4. P1c‐deficiency affects neurite branching and growth cone morphology in hippocampal neurons of WT and P0 mice. Table S1. Primary antibodies used in this study. Video Clip S1. Time‐lapse images of EB3‐mCherry comets in a WT DRG neuron. Video Clip S2. Time‐lapse images of EB3‐mCherry comets in a P1c−/− DRG neuron. Video Clip S3. Time‐lapse images of vesicles in WT DRG neurons. Video Clip S4. Time‐lapse images of vesicles in P1c−/− DRG neurons. Video Clip S5. Time‐lapse images visualizing MitoTracker‐labelled mitochondria in DRG neurons isolated from 3 month‐old WT mice. Video Clip S6. Time‐lapse images visualizing MitoTracker‐labelled mitochondria in DRG neurons isolated from 3 month‐old P1c−/− mice. Video Clip S7. Time‐lapse images visualizing MitoTracker‐labelled mitochondria in DRG neurons isolated from 3 month‐old P1b−/− mice. [file NAN-47-73-s001.zip › nan12635-sup-0003-FigureS3.tif]

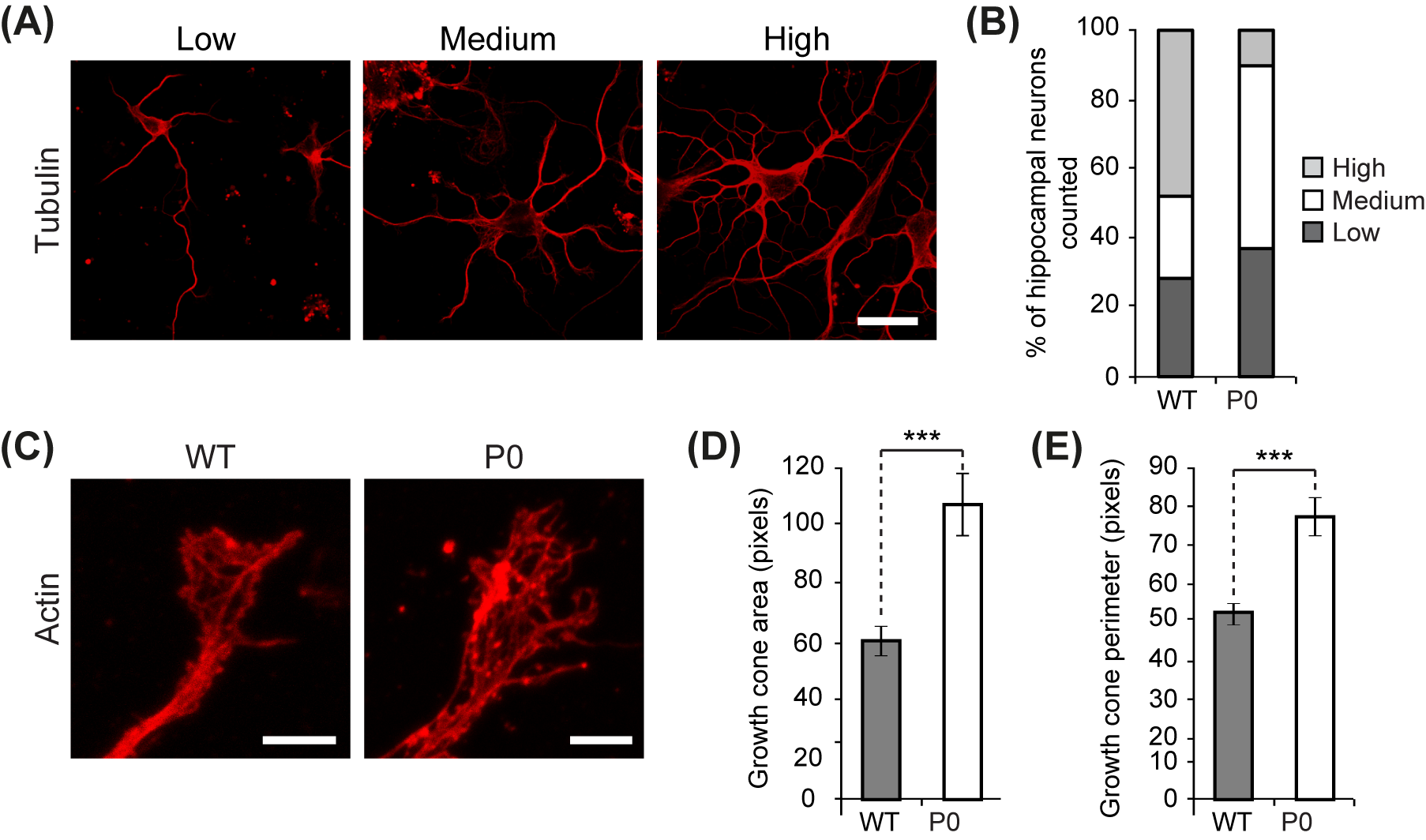

Supplement: Supplementary file 1 — Figure S1. Plectin‐dependent association of microtubule‐associated proteins (MAPs) with neuronal MTs assessed by co‐fractionation. Figure S2. Relative tau/tubulin signal intensities measured in hippocampal neurons of WT and P0 mice. Figure S3. Acetylation state of axonal MTs in hippocampal neurons of WT and P0 mice. Figure S4. P1c‐deficiency affects neurite branching and growth cone morphology in hippocampal neurons of WT and P0 mice. Table S1. Primary antibodies used in this study. Video Clip S1. Time‐lapse images of EB3‐mCherry comets in a WT DRG neuron. Video Clip S2. Time‐lapse images of EB3‐mCherry comets in a P1c−/− DRG neuron. Video Clip S3. Time‐lapse images of vesicles in WT DRG neurons. Video Clip S4. Time‐lapse images of vesicles in P1c−/− DRG neurons. Video Clip S5. Time‐lapse images visualizing MitoTracker‐labelled mitochondria in DRG neurons isolated from 3 month‐old WT mice. Video Clip S6. Time‐lapse images visualizing MitoTracker‐labelled mitochondria in DRG neurons isolated from 3 month‐old P1c−/− mice. Video Clip S7. Time‐lapse images visualizing MitoTracker‐labelled mitochondria in DRG neurons isolated from 3 month‐old P1b−/− mice. [file NAN-47-73-s001.zip › nan12635-sup-0004-FigureS4.tif]
